# Adapting Ourselves, Instead of the Environment: An Inquiry into Human Enhancement for Function and Beyond

**DOI:** 10.1007/s12124-023-09797-6

**Published:** 2023-08-19

**Authors:** Niklas Alexander Döbler, Claus-Christian Carbon

**Affiliations:** 1 Department for General Psychology and Methodology, University of Bamberg, Bamberg, Germany; 2 Research group EPÆG (Ergonomics, Psychological Æsthetics, Gestalt), Bamberg, Germany; 3 Bamberg Graduate School of Affective and Cognitive Sciences (BaGrACS), Bamberg, Germany

**Keywords:** Embodiment, Ethics, Human Enhancement, Human-Technology Relationship, Material Engagement, Postphenomenology

## Abstract

Technology enables humans not only to adapt their environment to their needs but also to modify themselves. Means of Human Enhancement — embodied technologies to improve the human body’s capabilities or to create a new one — are the designated means of adapting ourselves instead of the environment. The debate about these technologies is typically fought on ethical soil. However, alarmist, utopian, and science fiction scenarios distract from the fact that Human Enhancement is a historical and pervasive phenomenon incorporated into many everyday practices. In the vein of disentangling conceptual difficulties, we claim that means of Human Enhancement are either physiologically or psychologically embodied, rendering the merging with the human user their most defining aspect. To fulfill its purpose, an enhancement must pass the test-in-the-world, i.e., assisting with effective engagement with a dynamic world. Even if failing in this regard: Human Enhancement is the fundamental and semi-targeted process of changing the users relationship with the world through the physical or psychological embodiment of a hitherto external object and/or change of one’s body. This can potentially change the notion of being human. Drawing on a rich body of theoretical and empirical literature, we aim to provide a nuanced analysis of the transformative nature of this phenomenon in close proximity to human practice. Stakeholders are invited to apply the theory presented here to interrogate their perspective on technology in general and Human Enhancement in particular.

## Introduction

Humans are apex manipulators. Although many species on Earth use tools (Bentley-Condit, 2010; Shumaker et al., 2011), humans and many of their ancestors have developed and practiced material engagement unprecedentedly (Ihde & Malafouris, 2019; Malafouris, 2013). We use technology to modify the environment for our purpose; we rely on it to survive. However, the disastrous effects of anthropogenic climate change make us all too painfully aware of the destructive power of this strategy. Adapting the environment to meet our short-term demands seriously threatens the requirements for human life on Earth.

Even if we successfully limit environmentally harmful technologies, the next technological threat to our human condition is supposedly just around the corner. The name of the game? *Human Enhancement* (see Fukuyama, 2006). A term that is often used to denote (biomedical) technologies meant to improve our abilities and eventually create “better” humans whose capabilities exceed a certain norm (Buchanan, 2011; Coeckelbergh, 2011, 2013; Hauskeller, 2013; President’s Council on Bioethics, 2003).

The debate on this issue is notoriously passionate. Proponents on the edge of the spectrum see Human Enhancement as a welcome opportunity to pave the way towards becoming trans- and posthuman (Bostrom, 2003a, b, 2013; More, 2011, 2013). On the contrary, critics of Human Enhancement and transhumanism fear potential backfiring and the eventual loss of what it means to be human (Agar, 2010; Browne & Clarke, 2020; Fukuyama, 2009; President’s Council on Bioethics, 2003; Thomas, 2022).

Typically fought on ethical soil ranging the from concrete to abstract, key figures of the Human Enhancement discourse are the so-called NBIC (Nanotechnology, Biotechnology, Information technology, and Cognitive science), whose transformative potential is acknowledged by proponents and critics (Dupuy, 2011; Roco & Bainbridge, 2002). However, Human Enhancement is not a futuristic issue. Instead, the technological transformation of our capabilities is a pervasive and common historical practice (Buchanan, 2011; Clark, 2003; Greely, 2006). Since their earliest ancestors, humans seemed to have used technologies to adapt their individual capabilities to imposed demands, eventually reshaping their ontogenetic and phylogenetic trajectories.

Directly concerned with human behavior and mind, Human Enhancement seems like an ideal phenomenon for psychology to investigate. Indeed, there is a rich body of literature on the concerns and motivations underlying Human Enhancement efforts (e.g., Hotze et al., 2011; Pew Research Center, 2016; Sample et al., 2020; Sattler et al., 2021; Sattler et al., 2014; Scheske and Schnall, 2012). But the latest literature neglects to acknowledge the intricate and multifaceted nature of the technologies employed in Human Enhancement and their transformative effects concerning various aspect of human capabilities. To effectively bridge this gap, developing a comprehensive and adaptable framework that encompasses various dimensions, including the social one, is crucial. This framework should allow for pragmatic and practical implementation while maintaining flexibility to accommodate diverse perspectives and evolving circumstances. In this way, we can analyze the impact of Human Enhancement on our engagement with the world.

Adopting the notion that moral actions and decisions are hybrid and emerge from the relationship between humans and concrete technologies, we must investigate the precise role these technologies play in human practices (Verbeek, 2014). Ethical assessment and transformational capabilities of technologies are inextricable. Since Human Enhancement is treated as a highly controversial issue, we believe that a practical framework – informed by psychology – must investigate fundamental behavioral principles alongside concrete empirical manifestations.

Starting from the premise that Human Enhancement is commonly practiced, we ask: *What are we actually doing, and how does this pervasive behavior influence our relationship with ourselves and the environment*?

## Our Approach

We identify a need to integrate empirical findings on Human Enhancement within a broader framework that emphasizes how the being of humans is embedded and shaped by a socio-material frame (Coeckelbergh, 2013; Verbeek, 2005). This frame comprises various means to augment human capabilities in specific ways. Seminal works like Clark (2003) paved the way for understanding the human being as a dynamic and flexible. Although we draw heavy inspiration from Clark’s line of argumentation, his book “Natural-born Cyborgs” did not establish an explicit link with Human Enhancement and its various means and was rather focused on the individual aspect of the embodiment of external things.

Nonetheless, we believe that the work by Clark is indispensable for understanding Human Enhancement. Thus, we revisit his argumentation on incorporating external things and the respective effects on the “soft self” as well as what this means for our understanding of being human (Clark, 2003). We further seek to enrich and apply his arguments in regard to novel theoretical and empirical insights. This way, we may be able to examine the functional aspect and behavioral kernel of Human Enhancement within human practice and engagement with the world.

For this purpose, we use a methodology leaning towards postphenomenology. Rather than treating technology as an abstract phenomenon, postphenomenology investigates how specific technologies shape the human experience and engagement with the world (Rosenberger & Verbeek, 2015). Pursuing a comprehensive understanding of the relations between things and humans, postphenomenology draws upon insights from empirical research and philosophical considerations. (Rosenberger & Verbeek, 2015). Considering that Human Enhancement technologies are employed in close proximity, if not within the human body, it is appropriate to draw from a school of thought that underscores the importance of embodiment (Aagaard, 2016) and the intricate interactions between humans and technology (Ihde, 1990; Verbeek, 2008). This way, we can explore how a distinct set of technologies influences and shapes human subjectivity and practices in a dynamic and technologized world (Rosenberger and Verbeek, 2015). Although some technologies employed within Human Enhancement may not be commonly discussed in postphenomenology, we believe its principle assumptions of technology-mediated human experience and altered relations to the world can still be useful, regardless of whether the means are pills or external tool.

Drawing from a broad scope of literature, including the philosophy of technology and empirical and theoretical psychology, we strive to overcome the blind spots that arise when examining Human Enhancement from solely one angle. To better situate this practice and associated means, we also link the term to technologies commonly not investigated under this label. By integrating these different perspectives, we can address the historical and present ramifications of Human Enhancement while also contemplating its future potential on different scales of social organization. This inclusive approach aims to formulate a framework that can be applied within the research and development process of emerging technologies. By considering unintended and transformative effects, this framework may provide a robust structure that incorporates careful evaluation and proactive measures to mitigate risks and promote ethical considerations.

## Disentangling the Phenomenon

Human Enhancement is said to suffer from conceptual confusion (Gyngell & Selgelid, 2016), starting with disagreement about the means of enhancement. Some authors focus on technology in general (e.g., Coeckelbergh, 2011, 2013; Danaher, 2014; DeGrazia, 2005), some on biomedical means employed through the respective science and technology (e.g., Buchanan, 2011; President’s Council on Bioethics, 2003), and some do not explicitly limit their definition to specific methods (e.g., Daniels, 2000; Juengst, 1998; Nagel, 2014; Savulescu et al., 2011)^1^. With “Enhancement,“ “Human Enhancement,“ and “Enhancement Technologies” often used interchangeably, the confusion of definitions compounds when terminologies are mixed. Furthermore, authors often discuss their understanding of Human Enhancement in great detail, making it difficult to extract a concise definition.

Still, the differing conceptualizations of Human Enhancement can provide distinct insights (Gyngell & Selgelid, 2016). Commencing with a dissection of the two words it comprises, our investigation prioritizes the experience and relationship-altering potential of Human Enhancement in close proximity to human practice. Throughout this paper, we use “Human Enhancement” to refer to the general phenomenon. We capitalize it to emphasize its conceptual nature, which encompasses a variety of historical, contemporary, and future technologies. The term “enhancement” (lowercase “e”) is reserved to denote particular technological means categorized under Human Enhancement.

### The Human and the Enhancement

Not concerned with extending our dog’s lifespan or increasing our goldfish’s cognitive abilities, the target of any Human Enhancement intervention is a fellow *Homo sapiens*^2^. This yields passionate ethical debates about human dignity (Bostrom, 2005; Kirchhoffer, 2017), human nature (Buchanan, 2009; Coeckelbergh, 2013; Lewens, 2012; Pugh et al., 2016; Ramsey, 2012), and the potential to overcome being human (Bostrom, 2013; Hansell & Grassie, 2011). The ethical and scientific importance of the issue stems from the alleged transformation of human subjects into objects: humans who make themselves. Due to the emotional nature of the human issue at heart, discussions may become polarized and focused on sensational and contentious examples (Jones, 2006).

In addition, the mentioned human, whose status quo is deemed undesirable, is a *living human being*. The intricacy of human life in all its socio-material richness may obfuscate the universal activity of living that applies to *Homo sapiens* as to any other instance of life: maintaining a lower state of entropy within an internal environment through the utilization of external energy to preserve thermodynamic disequilibrium (Irwin & Schulze-Makuch, 2020). Governed by fundamental chemical and physical laws, the indispensability of this teleonomic activity is rooted in the complex and ever-changing interplay between internal and external demands (Pross, 2012).

However, as psychologists, our primary focus lies on the tangible manifestations of human behavior in everyday settings and the subjective experiences that individuals form during their activity of living. That is, we acknowledge that humans do not exist independently from the world. Instead, their being is a *being-in-the-world* (Heidegger, 1927/1967). Tied to the bodily and environmental characteristics that allow for survival, the direct awareness of human behavior, thinking, and existence shape and constitute the different spheres in which we must manage our individual lives. However, this conscious contemplation is only secondary to the more common pre-reflective everyday engagement with the world (Winograd & Flores, 1986). This understanding of our being dissolves the separation between thinking and acting, emphasizing how cognition refers to a pattern of adaptive behavior relevant to the organism’s functioning (Winograd & Flores, 1986). Through the historical structural coupling of reciprocal interactions between organisms and the environment, a distinct world is brought forward for humans to inhabit (Gallagher, 2017). Actively engaged in this world, its dynamic environmental properties and social processes can be a source of experienced unease or risk, calling for change and transformation (Coeckelbergh, 2013). In navigating these multifaceted challenges, we must acknowledge the bidirectional interaction between our mind and engagement with the material environment (Malafouris, 2013), highlighting how altering our environment is an effective and influential adaptive strategy (Kirsh, 1996).

The intentional manipulation of the environment can result in the skillful crafting of diverse material artifacts. Some of which are directly tied to Human Enhancement. Yet, in Human Enhancement, external factors are not intended to change but, the individual’s condition is the target for alteration. *Instead of adapting the environment to our demands, we are adapting ourselves.* Thrown into a world with perpetually changing demands, unpredictable events, and processes beyond our control, *being-in-the-(dynamic)-world* is constantly *adapting-(to)-the-world*, and Human Enhancement is part of the technological answer to this need.

In its broadest connotation, enhancements are a deliberate attempt to improve a specific feature (Buchanan, 2011), usually one that can aid in dealing with the aforementioned unfavorable circumstances. The enhancement reveals a non-desirable condition that shall be controlled to meet the world’s or, not to forget, our own demands. If embedded in a larger practice, the underlying motivation may be the desire to reliably improve the human condition as a whole (Bailey, 2014; Hauskeller, 2013).

The means to improve and adapt the human condition are manifold. To live longer, one could change their diet, quit smoking, or work out. Enhanced memory capacity may result from the application of mnemonic techniques. Numerous “historical” or “conventional” means of improving human capacities exist that are not subject to the same level of scrutiny as the more contentious cases of Human Enhancement (Bostrom & Roache, 2008; Bostrom & Sandberg, 2009; Buchanan, 2011; Caviola & Faber, 2015; Döbler & Carbon, 2021; Greely, 2006; Menuz et al., 2013). The profound influence of cultural advancements like literacy and agriculture on human skills and biology raises questions about the blanket moral condemnation of technologies that produce comparable outcomes through direct body modification (Buchanan, 2011).

While we concur on the ubiquitous and historical character of means to improve the human condition, we do not claim that any intervention intending to improve is Human Enhancement, but only those meant to be carried out by an embodied technology. This specification functions as an intelligible, morally relevant, and non-arbitrary line needed to employ our definition for ethical purposes (see Bostrom and Savulescu, 2009). It is employed to emphasize the practice of adapting *ourselves* instead of the environment and the respective link to the phenomenological effects revolving around the technologies we identify as means for Human Enhancement. Other methods to improve the human condition are not denied in their effect but are classified as different phenomena.

### The Technological Enhancement

The human lifeworld is technological, as is how we live in it (Allenby & Sarewitz, 2011; Coeckelbergh, 2013, 2018; Haff, 2014; Ihde, 1990). We are *Homo Faber*: We make to live, and while we live, made things eventually make us (Ihde & Malafouris, 2019). Human activity, perception, and experience within the lifeworld are mediated and altered through technology (Döbler & Bartnik, 2022; Ihde, 1979, 1990, 2010, 2011; Malafouris, 2013; Verbeek, 2005, 2008). Technology has extended and created human capabilities since the day our ancestors picked up a natural item and used it as *Tool Zero*. Hence, Human Enhancement is just another manifestation of the practice of exerting control over unfavorable circumstances through technological means.

When speaking of means, it is essential to distinguish between *technique* and *technology*. Techniques can be culturally transmitted and enhance human capabilities (e.g., mnemonics). When employed to improve human capabilities, they may be called “*enhancement techniques”* (Brunyé et al., 2020, p. 457). Technology, however, is a product rooted in skillful material engagement and the manipulation of the environment – something to achieve a goal and which serves a purpose (Allenby & Sarewitz, 2011; Carroll, 2017; Malafouris, 2013)^3^.

#### Embodied Technology

Although initially separate from the human body, technology can eventually merge with and impact it significantly. The means of Human Enhancement are initially but not sufficiently described as “embodied artifacts:” things that can only exercise their function when interacting with a human body (see Heersmink, 2021). However, Human Enhancement encompasses the application of external artifacts and advanced biotechnology operating within the human body, often beyond our ability to regulate its function once administered.

Capable of merging with the human body and mind means for Humane Enhancement are *embodied technologies*. From a *physiological* perspective, this is the case when enhancement drugs are metabolized or devices are implanted. Implementing exogenous components to adapt to a new and potentially hostile environment was the initial idea behind the “Cyborg” (Clynes & Kline, 1960), a term that has been subsequently adapted and extended. Picking up this idea, the term “Natural born cyborg,“ introduced by Clark (2003), illustrates how humans can effortlessly integrate different tools and objects into their body representation and cognitive processes. Clark describes what we call the *psychological embodiment* dimension of Human Enhancement: Tools that feel like being part of ourselves (Greely, 2006), become embodied (Ihde, 1990; Vignemont, 2011) and eventually nearly phenomenologically transparent (Clark, 2003, 2007; Ihde, 1990). Empirical evidence on tool embodiment and the flexibility of our body representation is plentiful (Holmes & Spence, 2004; Lin et al., 2022; Martel et al., 2016; Weser & Proffitt, 2021), suggesting that humans are indeed “profoundly embodied agents,” who are constantly renegotiating the boundary between them and their environment (Clark, 2007, p. 268)^4^.

Both kinds of embodiment – physiological and psychological – require somewhat spatial proximity of artifact and human. The psychological embodiment can best be understood under neurophenomenological terms, while the physiological dimension also introduces the dimension of invasiveness to the equation. An aspect that contributes to ethical suspicion (Allhoff et al., 2010; Haslam et al., 2021; Sattler & Pietralla, 2022; Scheske & Schnall, 2012).

With physical artifacts possibly integrated into psychological self-representation, the line between these two embodiment types may be blurred. At the same time, a primary psychological embodied artifact can also significantly influence physiological processes. Depending on the properties of the enhancement and its subsequent type of embodiment, the relevant human-technology relationship is somewhere located on the continuum between an embodiment relation (Ihde, 1990) (technology phenomenological incorporated – more psychologically embodied) and the more physiologically embodied cyborg relation (Verbeek, 2008, 2011), in which human and technology physically merge^5^. Within either of these relations, technology profoundly mediates and alters human intentionality and experience (Ihde, 1990; Verbeek, 2008, 2011), rendering the relationship and the constituting technology *intimate*.

Some combinations of these different extents of embodiment eventually lead to a sense of ownership (Vignemont, 2011). The type and depth of embodiment depend on how much the spatial, motoric, and affective measurements of embodiment indicate that the enhancement is approximatly “[…] processed in the same way as the properties of one’s body” (Vignemont, 2011, p. 84). According to Vignemont, we must also distinguish between the sense of ownership and the judgment of ownership. The former describes the immediate experience of something belonging to one’s body, while the latter refers to a descriptive conclusion about the same fact. Whether an enhancement can be sensed and judged as belonging to the body depends on the characteristics of the enhancement, which also influence the nature and degree of its embodiment. Since only an individual’s limbs may satisfy all the requirements for full embodiment (Vignemont, 2011), enhancements do not need to be processed on the same level as natural biological components of the human body; a close approximation should suffice.

Ihde (1990) reveals the inherent contradictions in seeking to experience the enhancing effects of embodied technology while remaining “unaware of its presence.“ Accordingly, the desire for transparency is elicited and constrained by the same means: “The user wants what the technology gives but does not want the limits, the transformations that a technologically extended body implies. There is a fundamental ambivalence toward the very human creation of our own earthly tools” (Ihde, 1990, p. 76). With humans notably striving for achievement, extended lifespan, enhanced cognitive capacity, and the transgression of established boundaries, a similar ambivalence emerges within the discourse of Human Enhancement. “Man is the only creature who refuses to be what he is” Camus (1951, p. 17) famously stated. Against this backdrop, Human Enhancement may be considered a viable tool of resistance. Nevertheless, these intimate technologies are not universally met with joyful anticipation. Embodied technologies highlight the fragility of the idea of an immaculate human body, which may contribute to the skepticism surrounding their use. While the embodiment of external technological and non-human entities may appear threatening to the the alleged “natural” boundary between humans and their external world, any reactionary countermeasures are ultimately defeated by the persistent and historical nature of Human Enhancement and the embodiment of external tools (see Clark, 2003). The embodied technology connects the Enhancement with the Human. It is the alleged solution and the potential problem.

By introducing new capabilities, the causal relationships within the dynamic system of brain, body, and environment can be substantially modified, resulting in a reciprocal transformation of its constituent elements. Therefore, any interference with the relationship between these components can affect information processing, with consequential effects on cognition (Gallagher, 2017). By enhancing our bodily characteristics and respective abilities, we can change our minds:

“practically any use of technology influences and changes the way the human mind functions, human enhancement technologies offer an even more radical possibility – that of adapting human beings directly to specific environments, niches and demands through the technological restructuring of the body, and especially of the brain.” (Pustovrh et al., 2018, p. 302)

This is also true for external technologies and tools, whose capacity to create novel agent-world circuits and – neurophenomenologically speaking – disappear upon fulfillment qualifies them as Human Enhancement (Clark, 2007).

Human Enhancement serves as a powerful demonstration of technology’s ability to mediate and constitutes relationships with the world and the subsequent experiences (see Verbeek, 2005, 2008). Tied to all sorts of technology, technological modification of our capabilities significantly influences our being-in-the-world (Coeckelbergh, 2013).

#### Human Enhancement in Practice

Increasing phenomenological transparency also occurs on a practical level. Once incorporated into concrete practice, an artifact is not consciously experienced anymore, yet mediates the relation of its user with the world. This and the transformation of human experience are byproducts of the technology’s functionality (Verbeek, 2005).

Mihailov et al. (2021) report how people deem cognitive enhancement less morally wrong if widely used. Peer behavior and experiences are valuable sources of information for considering whether to engage with an enhancement (Sattler et al., 2021). The disruptive potential of Human Enhancement seems to be problematic only insofar as enhancements remain external to established practices. Treating Human Enhancement as a purely futuristic and dangerous phenomenon may stem from the fact that some emerging and hypothetical technologies have yet to be assimilated into concrete practices. From a historical perspective, most Human Enhancement means have already disappeared. Not in the sense that it ceased to exist, but its transformative aspect is taken for granted as the natural way the world appears to us. Our *being-in-the-world* is already fleshed out and shaped by technologies, including Human Enhancement (Coeckelbergh, 2013).

When enhancements are indispensable to an activity commonly used for enhancement objectives, such an activity with its interrelated technologies and agents situated within a broader socio-material context can be classified as a *Human Enhancement practice*. Human Enhancement practices can profoundly change human capabilities and constitute a new relationship with the world. They emphasize how multiple enhancements can be absorbed by and embedded in human activity and how the sociocultural and reciprocal influence of the practice’s contingencies and components eventually shapes our minds and lives (see Coeckelbergh, 2018; Ihde and Malafouris, 2019; Malafouris, 2013; Verbeek, 2005).

The intended outcome of a Human Enhancement practice cannot be primarily attributed to a single technology but rather to the complex socio-material network of artifacts and techniques immediately at work during the practice^6^. Any instance of Human Enhancement has the potential to become part of a practice, which is also utilized for adaptation purposes and may give rise to familiar ethical controversies and effects. Adapting ourselves through Human Enhancement and Human Enhancement, in general, are practices themselves. If needed, they may be further segmented into individual practices and means, given that they involve modifying humans to fit their environment rather than modifying the environment to fit humans. Assembling different components, Human Enhancement practices occupy a higher hierarchical level of human socio-materiality than means of Human Enhancement or enhancement techniques.

### A New Definition

**Fig. 1:**
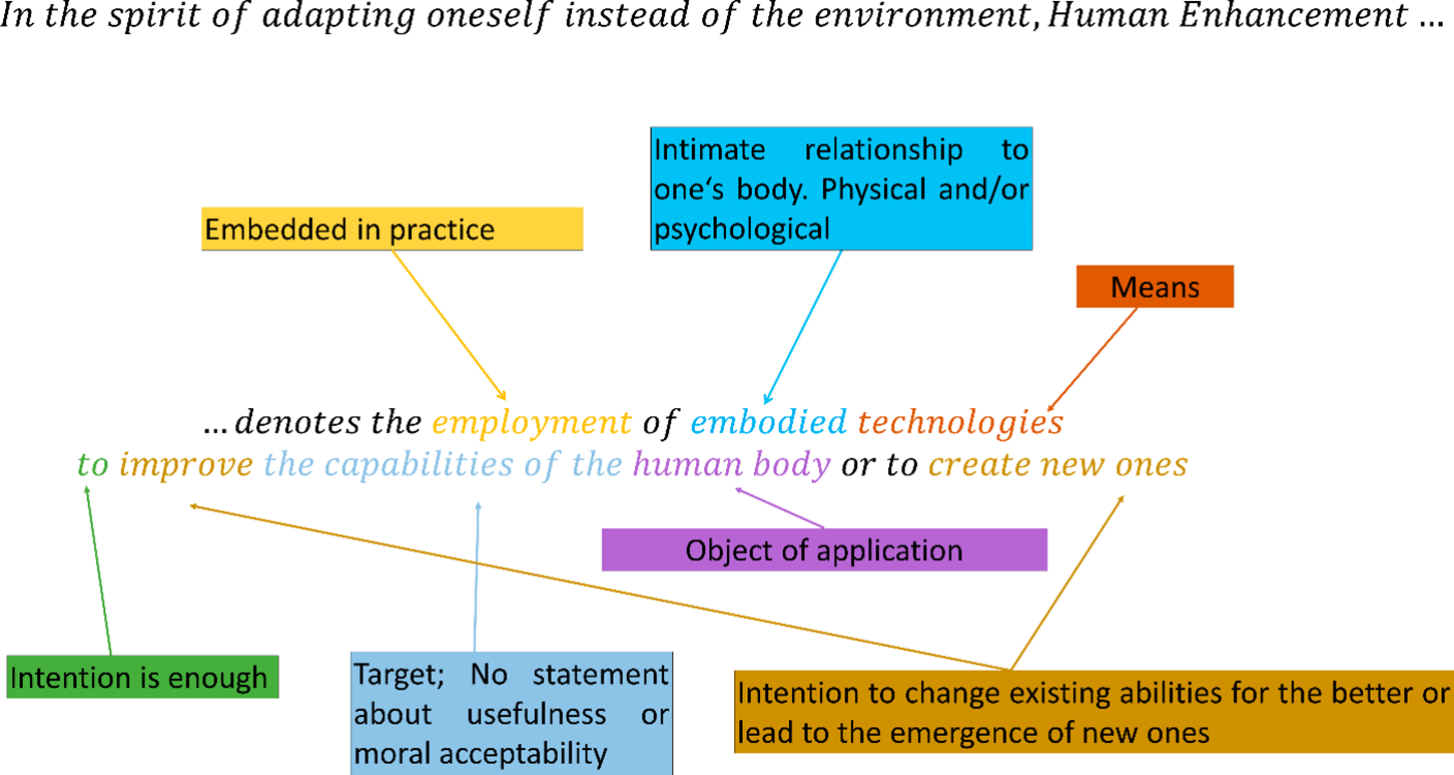
Our proposed definition of Human Enhancement. Colors were added to represent the different aspects. Boxes provide more context. Human Enhancement is not solely about enhancing or creating specific abilities. It is a semi-targeted and fundamental effort to alter the user’s relationship with the world by physically and/or psychologically embodying previously external objects or changing the body. This transformation leads to the potential for novel and transformed experiences that recursively modify the human-environment relationship. Subsequently, the user’s being, influenced by the same relationship, can be transformed.

Our approach attempts to emphasize the manifoldness of Human Enhancements, its timeless character, and how it is practiced by utilizing the human body’s ability to incorporate external things to achieve a better fit between individual capabilities and imposed demands or create an individual state whose realization was not afforded through environmental manipulation alone.

Hence, we define Human Enhancement as *the employment of embodied technologies to improve the capabilities of the human body or to create new ones. An endeavor primarily conducted to adapt oneself instead of the environment*.

**Fig. 2:**
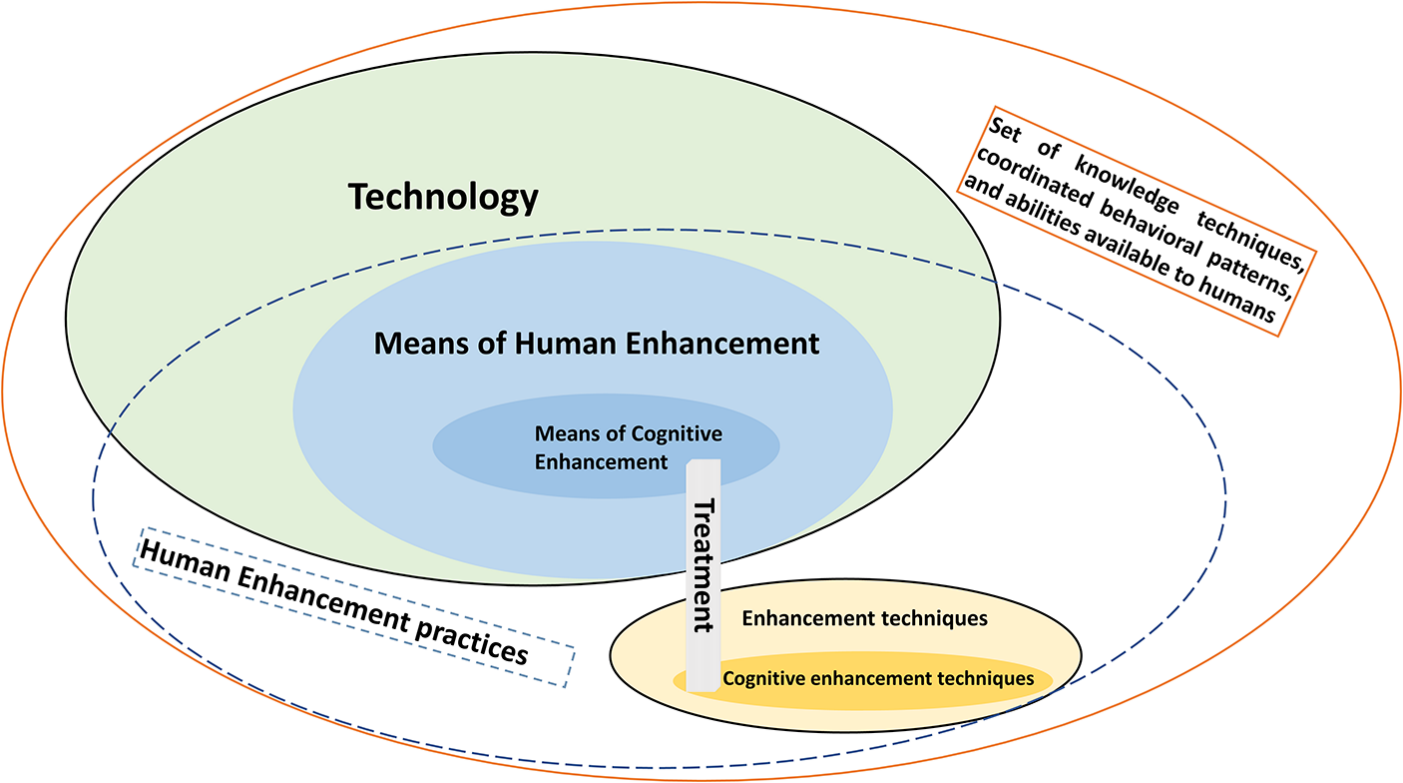
Conceptual overlapping of terms discussed here (Ellipsoids are not for scale). E.g., Cognitive Enhancement is Human Enhancement aimed at improving cognitive abilities. Treatment refers to interventions aimed at ameliorating an adverse bodily or mental condition

Figure 1 shows the definition and key concepts, which were or will be elaborated further. Figure 2 illustrates the interconnections between the proposed definition and other concepts we postulated, thereby facilitating the positioning of our perspective into the broader discourse. It particularly highlights the domain of cognitive enhancement, which represents a prominent area of empirical investigation (e.g., Becker et al., 2022; Racine et al., 2021), while also addressing the contentious issue of distinguishing it from the concept of treatment, a matter that frequently engenders controversial discussions (e.g., Bostrom and Roache, 2008; Hofmann, 2017).

There is a tendency to consider invasive and visually apparent artifacts as the primary instances of Human Enhancement (Döbler & Carbon, 2021). However, our definition expands beyond these examples and encompasses commonplace embodied tools and artifacts, such as clothing or a hammer, if they meet our definitory criteria.

Our definition can be classified as a “functional approach,” which emphasizes the capacity-improving aspect of Human Enhancement (Gyngell & Selgelid, 2016). Gyngell and Selgelid posit that functional approaches are often overly descriptive. Indeed, our definition does not make any normative statement about whether the enhancement will be good or bad. However, the normative value of any technology – and therefore also of any Human Enhancement – can be assessed in terms of the possibilities of action the respective technology provides in a specific environment to a specific user (Döbler & Bartnik, 2022; Klenk, 2021; Tollon, 2021). Moreover, as discussed later, evaluation of the enhancement hinges on its ability to foster effective engagement with the world and present normativity. By restricting the scope of Human Enhancement to embodied technologies and evaluating the moral value of specific enhancements based on their ability to facilitate actions and mediate human experience, our definition can be utilized for the identification and targeted moral assessment purposes.

We also acknowledge the emergence of grey regions of identification. Technologies in these areas are not necessarily more morally “grey.” Unless we adopt the notion that enhancements themselves are problematic, there is no reason to judge the ethical value of technology on an enhancement/no-enhancement dichotomy (Daniels, 2000). Thus, the moral evaluation and effects of Human Enhancement must be understood as existing along a continuum rather than being reduced to a binary categorization. Besides this “qualification” dimension, a different one concerns the ethical aspect. The latter can also be usefully addressed in terms of how technologies shape and mediate moral decisions and our engagement with the world (Verbeek, 2011, 2014). But at the same time, the potential for transformation is a function of the human-technology relationship (Ihde, 1990; Verbeek, 2008) in relation to the environment. Despite the need for contextualized moral judgments, certain human-technology relationships may carry more ethical significance, primarily due to their transformative potential in a given context.

Gyngell and Selgelid (2016) also argue that functionalist approaches to Human Enhancement tend to overlook the fact that enhancement can be achieved not only by improving existing capabilities but also by creating new ones, as well as through the deterioration of a physical condition. Our definition considers both aspects while acknowledging the variability in the exact enhancement mechanisms^7^ (see Chadwick, 2009; Menuz et al., 2013).

Functionalist approaches reject the treatment-enhancement distinction. We concur because the line between “healthy/sick” and “normal/unnormal” is partially drawn artificially (Clausen, 2009; Gee, 2016; Hauskeller, 2009; Hofmann, 2017; Lee, 2016; Lewens, 2012; Menuz et al., 2013) and individual abilities fluctuate depending on situation and age (Andersen-Ranberg et al., 1999; Bostrom & Roache, 2008; Salthouse, 2012). Furthermore, individual baseline and comparison group matter (Cassioli and Balconi, 2022). A cochlear implant (CI), for example, is undoubtedly an improvement in normal hearing function compared to other people with hearing impairments (Boisvert et al., 2020; Bond et al., 2009; Clausen, 2009). However, concerning the grand mean of the overall population, CI-users’ hearing capabilities might still be below the average^8^. Noteworthy, CIs are perceived as a typical example of Human Enhancement (Döbler & Carbon, 2021). Within our approach, technologies commonly employed for therapeutical reasons and are embodied can be means of Human Enhancement when they are employed in the same function: to adapt someone to imposed and perceived demands.

We acknowledge that individual or populational baselines can influence the degree of enhancement (Cassioli & Balconi, 2022)^9^. Still, our definition builds on the specific function of adaptation. Altering one’s diet, receiving physical or psychological therapy, and other similar practices operate similarly, but they are considered enhancement techniques or part of a Human Enhancement practice because they lack a primary and singular technological agent.

Allhoff et al. (2010) argue that only “internal” and physically integrated technologies qualify as Human Enhancement. They justify this claim of what we would call physiological embodiment with permanent access to these technologies. While the invasiveness of specific enhancements has significant ethical and usage-related implications (Haslam et al., 2021; Ireni-Saban & Sherman, 2021; Kostick-Quenet et al., 2022; Sattler & Pietralla, 2022; Scheske & Schnall, 2012; Whitman et al., 2018), the concept of Human Enhancement extends the limits of the skin. External technologies such as transcranial magnetic stimulation (TMS) or transcranial direct current stimulation (tDCS) are capable of (temporarily) enhancing performance-related abilities by altering internal bodily processes (Kim et al., 2019; Luber & Lisanby, 2014; Okano et al., 2015; Patel et al., 2021), and are thus physiologically embodied.

While some interventions are literally and physiologically embodied as they function inside the visible boundaries of the human body or directly act upon its capabilities, others, albeit external, are still embodied but more psychologically. The extent of this psychological integration into our body representation depends on the capacity to provide sensorimotor feedback and its integration into our cognitive, motoric, and affective action-response model of ourselves and our environment (Clark, 2007; Martel et al., 2016; Vignemont, 2011). The potential of external tools for Human Enhancement hinges on their ability to establish an effective agent-tool interface (Clark, 2007). Limiting Human Enhancement to internal technologies ignores the neurophenomenological reality of human material engagement, tool embodiment, and the experienced and evident notion of a “soft self” (see Clark, 2003).

After incorporating sensory feedback and the eventual formation of a sense of ownership (Vignemont, 2011), the embodiment of external objects creates a unique relationship with the world. As there are short and long-term types of embodiment (Vignemont, 2011), these effects may only be observable when the external tool directly interacts with the human body. Although other external technologies may generate embodied experiences, they lack the same level of integration and sense of ownership. That is, a thermometer hanging on the wall does not fall under our notion of Human Enhancement, albeit it brings forth new experiences and a different relationship with the world (see Ihde, 1990).

Technological developments do not merely alter our physical environment but also our ways of seeing, acting, and valuing. They are shaped by human agency and social forces and, in turn, shape our practices and social relations. (Carroll, 2017; Döbler & Bartnik, 2022; Dobres & Hoffman, 1994; Ihde, 1990; Ihde & Malafouris, 2019; Malafouris, 2013; Verbeek, 2005, 2011). The relation-altering potential of technologies, including instances of Human Enhancement, profoundly impacts how we interact with the socio-material environment (Döbler & Bartnik, 2022). Human Enhancement is not solely about enhancing or creating specific abilities. *It is a semi-targeted and fundamental effort to alter the user’s relationship with the world by physically and/or psychologically embodying previously external objects or changing the body. This transformation leads to the potential for novel and transformed experiences that recursively modify the human-environment relationship. Subsequently, the user’s being, influenced by the same relationship, can be transformed.*

Human Enhancement dissolves the thin line between humans and technology. It counteracts the modernist impression of a radical separation of humans and technologies, which obfuscates the pivotal role technologies play in shaping and conducting human practices (Verbeek, 2014). In Human Enhancement, the effect of technology becomes embodied. Rather than adapting the environment to our demands (Kirsh, 1996), we adapt the human body and mind (Pustovrh et al., 2018). Through Human Enhancement, we become the things we made.

## Inquiring Human Enhancement

As Coeckelbergh (2013) has put it, our human being consists of the inseparable cultural, social, and technological aspects of our condition that constitute our experience of the world. Some view augmenting human capacities as an opportunity to explore and discover new modes of being (Bostrom, 2003b, 2013). Others fear the widespread application of radical forms of Human Enhancement will alienate us from ourselves, the world, and our unenhanced peers (Agar, 2010; Dupuy, 2011; Kass, 2003; President’s Council on Bioethics, 2003). However, even the proponents of this view admit, that an abrupt loss of humanity as a value is unlikely (Agar, 2014). Still, the perspectives of both advocates and critics converge on the understanding that Human Enhancement, by changing us, can alter our relationship with the world.

We claim that the fundamental principle of adapting ourselves instead of the environment has ramified into the development and adoption of various means of Human Enhancement. These means and surrounding practices come with particular transformative effects we seek to elucidate.

To assess Human Enhancement from a functional and ethical standpoint, we will examine various aspects and conclude each section with specific questions. These questions aim to aid the practical implementation of our ideas and assist stakeholders in predicting and evaluating the transformative and ethical effects of technologies that may qualify as enhancements. Human Enhancement has the potential to, and as we will argue later, have already impacted our fundamental understanding of what it means to be human. This renders conducting a careful investigation into the concept indispensable. Assessing the tangible effects of various means for Human Enhancement allows us to avoid the pitfall of reducing these technologies to their conditions of possibility (e.g., a perceived mismatch between demands and capabilities + embodiment) (see Verbeek, 2005).

Instead of an utterly skeptical or careless Laissez-faire approach, we call for a thorough case-by-case examination. Acknowledging Human Enhancements’ manifoldness and effects, we refrain from adopting a categorical ethical rejection of it. That is, the focus of our inquiry is not the aspect of improvement per se but how the intended improvement of a specific enhancement will change our understanding of ourselves, our world, and our existence within.

### Is it Always an Improvement?

The matter of improvement carries important implications (Chadwick, 2009). When assessing it, two factors demand particular attention: task-specificity and side effects.

The first question concerns the application width of the Enhancement. Is the underlying intervention designed to serve a specific goal in a limited task, or is it meant to be a more general improvement? Examples of the former are the use of genetic engineering technologies to enhance radiation resistance during long-term space missions (Szocik et al., 2020a; Szocik et al., 2020b), vaccination against a specific disease (Döbler & Carbon, 2021) or sports engineering and cognitive doping in elite sports (James, 2010; Mihailov & Savulescu, 2018). The role of application context has been shown to influence acceptance of (cognitive) enhancement (Conrad et al., 2019; Hotze et al., 2011; Mihailov & Savulescu, 2018; Sample et al., 2022; Sattler et al., 2022). Furthermore, certain occupations may require Human Enhancement (Bickford, 2019; Grewal et al., 2020; Pustovrh et al., 2018). Generally, enhancements meant to be very task-specific are well-researchable and allow for a more straightforward developmental process.

Whereas specific enhancements tend to be characterized by a clearly defined purpose, general enhancements are typically represented by unspecific, optimistic aspirations for improved quality of life, cognition, or longevity, underpinned by the emphasis that individuals should be given maximum latitude in exploiting the possibilities presented by enhancements (Bostrom, 2003b, 2013; Sandberg, 2013). Pursuing a comprehensive improvement through a single intervention seems untenable unless it is suspected to impact a condition with an unequivocal beneficial value positively. The broader ’better humans through technology’ (Coeckelbergh, 2011) seems more of an academic perspective operating with abstract concepts like “humanity” and “better”. Hence it may be less suitable for examining the tangible effect of individual Human Enhancement. Yet, different scopes of analysis are needed. Task-specific enhancements can have vast consequences, which must be discussed in a broader context. Vaccinations, for instance, are very task-specific. Nevertheless, their impact can spread from the individual to the societal level (Bärnighausen et al., 2014). In addition, attitude toward this particular enhancement may be grounded on less task-specific but fundamental beliefs about agency, naturalness, and health (Browne, 2018; Döbler & Carbon, 2021). Individual enhancement effects may be minor, but if successfully and widely adopted, small and specific effects may lead to a cumulative enhancement effect and a positional augmentation of humanity’s capacity baseline (Cassioli and Balconi, 2022). However, effect accumulation may lead to an unpredicted trajectory and severe side effects on higher levels of systemic organization (Allenby & Sarewitz, 2011).

The literature has extensively discussed the potential negative consequences of abstract enhancement efforts (Agar, 2010; Bailey, 2014; Danaher, 2014; Hauskeller, 2013; President’s Council on Bioethics, 2003). Are longer lives good? When is faster cognition beneficial? And should we really be able to control every aspect of our lives? While undoubtedly valuable and vital, these discussions are situated on a more abstract, philosophical level.

Concerning individual and more tangible effects, people are more reluctant to (cognitive) enhancement if accompanied by severe side effects or if considered unsafe (Conrad et al., 2019; Kostick-Quenet et al., 2022; Sattler et al., 2013, 2014), medical professionals are less likely to prescribe unsafe enhancements (Hotze et al., 2011; Ram et al., 2021), and interventions that tangibly enhance human capabilities such as TMS, deep brain stimulation, and caffeine can have adverse bodily effects or risks (Kantzanou et al., 2021; Pallarés et al., 2013; Patel et al., 2021). Approaching side effects from a phenomenological angle, different technology-human relations can amplify or reduce certain aspects of human experience (Ihde, 1979, 1990), leading to the unwanted and unforeseen transformation of human experience and values. An unwanted acceleration of this phenomenon may support the related concerns about radical enhancement (Agar, 2014) and underscores the need to evaluate both instrumental and experience-related outcomes (Cassioli & Balconi, 2022).

Disentangling the complex discussion of side effects of Human Enhancement, we must always assess its *horizontal dimension*: The enhancement of a specific capability may be accompanied by deteriorations or improvements in different ones (Bostrom & Sandberg, 2009; Caviola & Faber, 2015; Davies, 2017; Hauskeller, 2013; Menuz et al., 2013). The ratio of desirable and tangible effects to undesirable side effects in a given context is probably one of the main factors in deciding whether to use an enhancement. Exclusively prioritizing predefined performance measures may miss important ethical considerations and transformative impacts. Therefore, we should ask: *In which context may the enhancement benefit its users, and what are possible individual (phenomenological) side-effects of the enhancement?*

### Testing Intention vs. Effect

Is a technology that has no improving or no effect at all an enhancement? Although it may be perceived as naïve, conceptualizations are indecisive. Some definitions mention only the intention to improve humans or do not explicitly state that the intervention must be successful (e.g., Brand et al., 2016; Buchanan, 2011; Coeckelbergh, 2011; Danaher, 2014; DeGrazia, 2005; Juengst, 1998; Menuz et al., 2013; Nagel, 2014; President’s Council on Bioethics, 2003). Others imply that only a measurable effect renders a technology an enhancement (e.g., Agar, 2014; Allhoff et al., 2010; Almeida and Diogo, 2019; Cassioli and Balconi, 2022; Coeckelbergh, 2013; Daniels, 2000; Gyngell and Selgelid, 2016).

The notion of objective improvement faces two challenges, namely the unpredictability of effects and the complexity of assessing how quantitative enhancements translate into qualitative improvements, considering all side effects (Chadwick, 2009). According to Chadwick, framing enhancement in terms of improvement requires to assess whether the purpose of the intervention is met. However, such evaluation may change rapidly and is contingent upon various factors. So in order to avoid an oscillating enhancement/no-enhancement distinction, should we subject ourselves to a purely quantitative notion that classifies enhancements by their ability to increase human capabilities objectively (Chadwick, 2009)? Doing so risks excluding futuristic transformative and controversial technologies that are often at the center of passionate ethical debates. If we cannot assess effects because the technologies in question do not exist and cannot be tested to determine whether their intention is met or if they have an effect at all, strictly speaking, any discourse referring to the label summarizing term Human Enhancement would be inadmissible: A discursive deadlock.

These problems may be solved once one adopts the notion that intention outweighs tangible outcomes when qualifying the application of a technology as Human Enhancement. So the “assumed functionality” (Wolff & Brand, 2013, p. 2) when employing enhancements to adapting ourselves instead of the environment is the defining component for identification. Contrary to other positions (Chadwick, 2009), we do not claim that the “improvement” concept should be ignored when defining Human Enhancement, but rather that it should be assessed in the context of intention, not as tangible outcome^10^.

#### A Success Story

When pursuing the enhancement of capabilities, people use various technologies solely based on subjective beliefs and often in direct contradiction to scientific insights. For example, they overestimate the tangible effects of cognitive enhancements (Ilieva et al., 2013) and the enhancement’s ability to target complex cognitive processes (Mihailov & Savulescu, 2018). Expectations are also easy to manipulate (Sansevere et al., 2022). Effects of cognitive enhancement drugs tend to be small and do not meet expected outcomes (Repantis et al., 2010; Roberts et al., 2020). People also seem not to opt for the most effective (i.e., illicit) option when pursuing instrumental drug-related goals (Brand et al., 2016), showcasing the possible constraints of individual enhancement efforts when moral and social laws regulate the most effective means.

The subjective component of Human Enhancement goes so far that people reported consuming alcohol to boost cognitive performance (Maier et al., 2018). Certain aspects of human consciousness can undoubtedly be enhanced by alcohol (Costa et al., 2021). But users actually reported decreased cognitive performance (Maier et al., 2018). Other technologies employed for enhancement purposes that show little to no tangible effect comprise exemplarily nutrition supplements (Crawford et al., 2020; Forbes et al., 2015), some over-the-counter neurotechnologies to boost cognitive performance (Wexler & Reiner, 2019), and drugs marketed as sexual enhancing but with little to no scientific evidence (Corazza et al., 2014). Although oversold, all these technologies have in common that they are employed to enhance and improve human functioning. Different from approaches that champion an outcome-focused a-posteriori perspective (e.g., Menuz et al., 2013), we want to emphasize the reciprocal connections between intention and outcome when coping with internal and external demands^11^. Hence, we call for a dynamic mixture of *a priori* and *a posteriori* assessment. We state that embodied technology is an enhancement if applied with the intention to enhance. One aspect of this intention is the belief that the technology will have a transformative effect and is a sufficient adaptation of oneself to imposed demands. Still, there is a difference between applying the enhancement and *being enhanced*. That is, beliefs about the sufficiency of the adaptation can only be maintained insofar as the improvement passes the *test-in-the-world.*

#### Test-in-the-world

The concept of *test-in-the-world* refers to the ongoing need to evaluate the effects of interventions in terms of meeting expectations, facilitating coping with imposed demands, and effective engagement with the world. This assessment is not necessarily dependent on scientific, “objective” inquiry but also rests on the subjective ability to effectively negotiate bodily capacities and dynamic challenges in the everyday engagement with the world.

Investigating human cognition and engagement with the world requires a hybrid approach that acknowledges the relationship between individual, biological, and cognitive processes and a pre-structured socio-material environment (Clark, 2013). Thus, we must reject an overly idiosyncratic approach and instead embrace an explanatory framework that integrates the embodied individual and the larger socio-material sphere that gave rise to various products of material engagement. In this vein, we approach what Ihde (1990) called *Micro-* and *Macroperception*. Accordingly, individual sensory and bodily (possibly technologically mediated) Micro-perception is always situated within the broader framework of the intertwined and overarching cultural-hermeneutic Macro-perception that was significantly shaped by technological mediation. The interrelation of these types of perception can be illustrated by every human-technology interchange (Ihde, 1990). This means that the individual perception and meaning of enhancement effects are always evaluated against and constituted by a broader cultural sphere (see Menuz et al., 2013).

At the micro level, a human organism employs a repertoire of skills and abilities to maintain structural integrity and thermodynamic disequilibrium in response to the dynamic demands of its environment (Irwin & Schulze-Makuch, 2020; Ramstead et al., 2016). For this task, accurate perception, effective cognition, and good predictive models are indispensable (Clark, 2013; Vernon et al., 2015). One abstract step forward, perception, action, cognition, and attention are interconnected neural mechanisms employed by life forms to perform the perpetual task of prediction error minimization (Clark, 2013). This error occurs when sensory input diverges from the neurophysiological prediction-generating model that the organism has constructed and adjusted through active participation in the world; a participation brought forward by the same model (Clark, 2013, 2019; Gallagher, 2017). Using sensorimotor skills is paramount when individuals negotiate the multifaceted relationship between them and the environment. This entails drawing upon the implicit body schema and its interconnected action capabilities to discern and execute suitable actions (Clark, 2007). The alteration of bodily states changes the salience-topography of the world and eventually yields novel information that influences future predictions (Clark, 2019). Hence, microperceptual processes are directly linked to the means of Human Enhancement as embodied technologies that transform human experience. One can certainly contemplate that a particular enhancement is no “natural” feature of one’s body. But pre-reflectively and seen from a neural point of view, this differentiation is resolved (Clark, 2003, 2007; Malafouris, 2013; Martel et al., 2016).

Depending on imposed demands, Human Enhancement can help choose the appropriate action, or may itself be the appropriate action. This action-selection process can be understood as the constant attempt to obtain an “optimal grip” on every situation (Bruineberg & Rietveld, 2014; Rietveld & Brouwers, 2017; Rietveld et al., 2018). Accordingly, organisms are selectively open to the relevant possibilities of action (affordances) the environment provides to minimize an experienced unfavorable internal disequilibrium. An improved grip can be understood as the successful minimization of prediction error by the adequate action-perception response to the dynamic field of multiple relevant affordances (Bruineberg & Rietveld, 2014; Ramstead et al., 2016). Note how this is linked to the notion of socially conveyed and individual-adopted pre-reflective normativity (Rietveld et al., 2018; Rietveld & Kiverstein, 2014), i.e., the cultural-hermeneutics that informs and structures individual perception (see Ihde, 1990).

Well aware of the limited translatability of basic neural mechanisms to higher levels of cognition, we believe our examples can elucidate the explanatory gap between the general principle of prediction error minimization and humans’ multifaceted adaptive strategies (see Clark, 2013). Overall, awareness of the precise functioning of lower-level cognitive functioning may not be necessary to explain conscious phenomena (Clark, 2019). Humans engage with their environment largely pre-reflectively. Still, they are able to consciously modulate their responsiveness to environmental solicitations (Dings, 2018). Modulated responsiveness will yield different actions, whose selection is informed by the relation between what is considered to be external and dynamic bodily states (Clark, 2019). Moreover, the ability of responsiveness modulation is connected to our diachronic culturally embedded existence, encompassing the manifold socio-material affordances, including their meaning and malleability (Dings, 2021). Considering this, we must focus on the historical, generative, and meaningful dimensions of human material engagement and its reciprocal influence on the human mind (Ihde & Malafouris, 2019; Malafouris, 2013). That is, human-technology interactions have been proven successful for effective world engagement, and prediction error minimization because cognitive function and tools are co-dependents (Malafouris, 2013) and because the rich landscape of affordances humans navigate so effortlessly is constituted by socio-material forces (Rietveld & Kiverstein, 2014), precisely to serve the overarching goal of effective engagement with the world (Clark, 2013). On a “higher” level of neural functioning, the very notion of the perceiving and acting subject is co-shaped by technology (Verbeek, 2005). But these more abstract cognitive abilities can only be made sense of against an intersubjectively shared backdrop of action and perception (Gallagher, 2017).

Our point here is rather conceptual than rigorously neuroscientific. We also want to stress how the basic notion of prediction *error* on a lower level of neural activity refers to some internal normativity that can be linked to the types of normativity expressed in higher levels of human social organization. At this level, distinguishing right from wrong is entangled in a web of dynamic values, situations, expectations, and conventions. For the nervous system, laws and habits may only exist as abstract internal physical activity, but this does not render these aspects neglectable. Instead, participation in the human lifeworld is largely regimented and scaffolded by the norms, conventions, and products of material engagement and their social-historic origin and use (Clark, 2013; Ihde & Malafouris, 2019; Malafouris, 2013; Rietveld & Kiverstein, 2014). Effective engagement with present normativity must be realized by the respective adequate behavioral outputs and the correlated neuronal activity. The reference norm is highly contextual. Adequate actions in one situation may be unacceptable in a different one. This expands even to the realm of non-ethical actions. Here, individual behavior may still somewhat align with a higher level of socially shared and conveyed normativity (Rietveld, 2008; Rietveld & Kiverstein, 2014). But inadequate actions when solving a puzzle do not have the same implications as violating the law. We must evaluate the test-in-the-world for effective engagement with situational and task-related demands and, if necessary, consider the alignment of outcomes with the sphere of moral evaluation.

The technological context, in all its different manifestations, from streetlight to genetic engineering, constitutes the macro-perceptional interpretational and shared backdrop (Ihde, 1990; Schmidl, 2022) and also determines what actually qualifies as (prediction) error. Generative models, which are believed to underlie every aspect of cognitive functioning, including consciousness, might achieve this status by becoming entangled with a diverse range of information from numerous sources. This entanglement is manifested by blending sensory patterns retrieved from “objective” features of the world with bodily states and responsiveness (Clark, 2019). Linking the micro and macro, the concept of the predictive brain seems to be a suitable explanatory companion for arguments that emphasize the embodied dimension of the human mind in relation to the environment (Clark, 2013). Approaches like Ihde’s install embodiment as a cornerstone of their theories, especially under the notion of pre-reflective integration of tools and things (Aagaard, 2016), which is vital to understand human technology-interaction. This way, our cognitive processes, heavily determining if we can effectively engage with the world in all its different spheres, are entangled with technology because the experience-transforming effect of technology affects the information that is used to update the generative model and its predictions.

#### Testing in Action

The agenda of control over the human condition (Hauskeller, 2013) can be seen as a cultural-hermeneutic, thus, macro-perceptual tending for an optimal grip. Emerging NBIC technologies have accelerated the controversy around Human Enhancement by promising more control over the current state of humanity, which advocates identify as undesirable disequilibrium. On the other hand, opponents of enhancement may assert that these enhancements disrupt or have disrupted the relation between the current human condition, a “natural” environment, and the historical trajectory of humanity, thereby generating the disequilibrium in the first place.

The need to enhance for survival is acknowledged by critics, who, in turn, focus on the more radical and more troublesome examples of Human Enhancement (Agar, 2014). From a historical perspective, rejecting Human Enhancement quite generally means risking losing the grip on our environment and eventually disintegrating. Note how the sense of presence or literally being in the world is constituted by our ability to act on behalf of our intentions (Triberti & Riva, 2015). Contrary to the fears of alienation from the world and ourselves (e.g., Agar, 2010; Kass, 2003; President’s Council on Bioethics, 2003), enhanced abilities may foster a tighter “interlocking” between individual intentions and the environment, resulting in an enhanced sense of presence and agency (see Triberti and Riva, 2015). It seems like the better our grip on the situation, the more we feel in the world. Even the immediate feeling of authenticity is suggested to result from a proper fit between the individual and their environment (Schmader & Sedikides, 2018). *Losing touch* with important aspects of the human condition is a relevant and literal concern when we think about virtual minds, wired brains, and dis-embodied agency (Agar, 2010; Hauskeller, 2013; Steinert et al., 2019; Žižek, 2021). But when becoming an interface, the embodied tool ceases to be a boundary and is rendered an instance of Human Enhancement (Clark, 2007; Malafouris, 2013).

As the embodiment of artifacts may require training and time (Heersmink, 2021; Kieliba et al., 2021; Weser & Proffitt, 2021), people must learn how to coordinate and integrate new capabilities in their functional model of the world and themselves. Expertise increases the feeling of tool embodiment (Weser & Proffitt, 2021), suggesting that the different degrees of embodiment (Vignemont, 2011) are also contingent on individual skills in relation to a challenging environment. This is further exemplified by Kieliba et al. (2021), who attached a third robotic thumb to the hand of healthy participants. After a five-day training period, users’ ability to employ the thumb in various tasks increased but remained below the control level. The additional thumb was experienced as embodied and led to a change in the hand’s neurological motor representation with which it was attached. Given the sub-control performance in the task, one could question the identification of this thumb as means of Human Enhancement. Yet, fulfilling its full potential may depend on sufficient training and specific tasks. Human Enhancement can also appear prima facie use- and effectless, but this evaluation may be contingent on situational and individual variables.

Unpredicted effects, which can interfere with the whole test, are possible. Failing the test-in-the-world provides an opportunity to reassess the efficacy of the enhancement and make necessary adjustments, either through a reversion or augmentation of the initial intervention. Moreover, a passed test is not definitive. What once promised a short-term performance boost might have severe side effects in a different situation. Once we have acted sufficiently to reduce the internal tension, the situation and its affordances may change, and the dynamic cycle of demands, adaptation, and testing begins anew. Changes of relevant affordances depend on agentic modification of the environment, agent-independent dynamic of the environment, and the interindividual dynamic of body and brain (Rietveld et al., 2018), but also due to a technologically induced change to the body’s capacities (Döbler & Bartnik, 2022). These strategies can be implemented at both the individual and societal levels and are more or less directly linked to the attempt to align capacities with demands, therefore also minimizing prediction error in a dynamic world (see Clark, 2013). Yet, the complex, intransparent, hard-to-predict, and dynamic socio-cultural environment functions as a challenging limitation for the one-to-one translation of enhancement intention and effect (Allenby & Sarewitz, 2011). Potential disastrous consequences are not exceptional to manipulating the environment but can also occur when we attempt to adapt ourselves technologically to imposed demands.

In the test-in-the-world paradigm, both the physical reality of the living system, with its action-perception loops and conditions that allow for the existence of the system, and the social reality governed by moral norms and conventions, must be considered. Bearing this necessity in mind, it becomes evident how the experience transforming abilities of technologies (Ihde, 1990), through the introduction of new agent-world circuits (Clark, 2007), connect the individual living system with the broader moral sphere and thus create a hybrid morality between things and humans, in which our ability to take outcome responsibility hinges on the recognition of the close connection between them (Verbeek, 2014). Human enhancement highlights this fact with unparalleled clarity.

On a physiological level, for instance in terms of genetic engineering to improve resistance against cosmic radiation (Szocik et al., 2020b), effective engagement with the world can be operationalized through health-related measurements. Tests can be more complicated if the goal is to improve cognitive skills, e.g., to increase exam performance. Still, all of these effects and their measurement are subjected to conscious micro-perceptual evaluation, but their interpretation always relates to the cultural-hermeneutic realm of macro-perception (Schmidl, 2022). The complexity of the spheres directly contributes to the dynamic of the test.

Depending on the context, external causal attribution of exam performance shifts between human and enhancement (Mihailov et al., 2021). Why would this principle not apply when an individual evaluates the impacts of enhancements? Disappointed expectations of the enhancement effect, for example, by failing an exam, may be attributed to factors other than the enhancement. As long as the individual holds to the subjective belief that their capacities were improved, they will experience an altered relationship with the world. This belief is conveyed by the emergence of different predictions on different levels of conscious accessibility. Translated to expectations, these predictions influence the perception of the possibilities of action the environment offers (Ramstead et al., 2016) and, thus, the ability to increase one’s grip on the situation (Rietveld et al., 2018). One conscious strategy to explain failed predictions are different causal attributions, a strategy often and successfully employed to reduce internal tension (Roesch & Weiner, 2001). From a neurophysiological perspective, this strategy may be understood as the modulation of hyper priors in the predictive model and the refinement of prior expectations to align with sensorimotor information, resulting in a fine-tuning of the overall processing (Clark, 2013; Clark et al., 2018).

Lack of awareness or denial of the technology’s enhancement potential (Döbler and Carbon, 2021) can lead to divergence between subjective and objective enhancement effects. However, even if enhanced individuals do not attribute an objective change to the enhancement, they may still experience a change in their capacities. The fact that they do not recognize the enhancement in its effect does not render the effect unrecognizable, nor does it eliminate a subtle pre-reflective influence on their perception and action. A person may think vaccination is poisonous and reject any application. But if vaccinated unknowingly, the person’s defense against the specific disease would still be enhanced because this technology assists with effective engagement with a world that hosts potential hazards. Likewise, individuals may use an ineffective substance to prevent infection, resulting in a perception of reduced pathogen danger despite lacking objective protection. Another example of unknown enhancement is the test of objective enhancement effects in randomized-placebo-control trials.

We encounter two perspectives here. First is the subjective evaluation. Is the enhancement working in the way I imagined? Do I feel like I can meet the demands imposed on me? If not, who or what is responsible? Secondly, the more objective assessment of the technologies effect: To what extent the transformed individual can react and act sufficiently on behalf of internal and external demands? A recent study on passive exoskeletons showed how positive objective and subjective task performance measures can concur (Maurice et al., 2020) and how the test is passed. In contrast, Gilbert et al. (2017) report how deep brain stimulation can lead to distorted perceptions of Parkinson’s disease patients regarding their abilities, meaning that the enhancement would not pass the test entirely in these cases. The assessment of enhancement effects, whether from subjective or objective viewpoints, is inherently bound to the larger context of macro-perception that situates the subjective experiences of individuals and the hermeneutic meaning of “objective” scientific measurements within a larger socially shared context (Ihde, 1990; Schmidl, 2022).

The evaluative authority of the social environment can go so far as to nullify the subjective dimension of the test effectively. The forceful application of Ritalin to a delinquent child for the sake of cooperative classroom behavior may medicalize social problems (Juengst, 1998), but from the perspective of the other agents, their engagement is improved, and the test passed.

On a macro level, the individual dynamic and affective process of tending towards an optimum grip manifests itself in the development and practice of manipulating the environment and adaptation of ourselves to the environment and imposed demands through Human Enhancement. The set of available strategies, practices, and technologies are contingent on the human ecological niche, which summarizes how we live (Gibson, 1979). These practices have somewhat proven to be effective in their application. Here, the mutual constitution between micro and macro level (Ihde, 1990) becomes visible. Context and individual foci may determine what counts as *effective*. Still, effective engagement is a category whose individual assessment is only possible if the normative conditions of effectiveness are conveyed on a macro level and can be tested against.

Further linking macro and micro levels, the individual body’s structure reflects the niche’s structure in a way that the individual can be attuned to the relevant affordances the niche consists of (Rietveld et al., 2018). As natural-born cyborgs (Clark, 2003), we are constantly engaged in embodied action (Gallagher, 2017) and can explore novel ways of relating to the world with technology and increase our grip. Means of Human Enhancement are a historical part of the human ecological niche simply because the niche is and was recursively shaped by the biological and neural ability to embody sticks, stones, and other external things (Clark, 2007) in a self-reflective way (Clark, 2003). The openness to incorporate futuristic and increasingly transformative technology goes so far that it can potentially dismantle its foundation. One of the most extreme and not-so-far-distant examples is using brain-computer interfaces to do things with thoughts: An embodied technology that enables its user to engage with the world in a *dis-embodied* manner (Steinert et al., 2019).

Under the notion of a dynamic human-environment relationship and the test-in-the-world, we state that an enhancement passes the test when it helps to achieve a better grip on the situation or, in short, assists with the effective engagement with the environment. This yields a difference between the application of an enhancement and *being enhanced*. Failure to pass the test does not change that the enhancement was usually applied out of an enhancement motivation^12^. Successful enhancement in terms of improvement, all side effects considered, may be linked to moral permissibility (Chadwick, 2009). Given the dynamic contextuality of the test-in-the-world across different timescales may significantly impede efforts to come to a definitive conclusion on this ground. We may also encounter a conflict between the different evaluative spheres for the test. Imagine using a strength-enhancing drug with no adverse physical side effects. Once applied, your lifting ability is raised by twenty pounds. Within the task the test is passed. However, if you use it in a competitive setting that prohibits such drugs, social evaluation may introduce another urging dimension for the test. Although the enhancement passes the test task-wise, it may fail to do so from an ethical perspective.

If the intention is first and objective improvement second, we must ask: *How do intention and effect align? Will the enhancement pass the test-in-the-world and enhance its user?* And: *What are the relevant dimensions of evaluation?*

### What About the Self?

The representation of the bodily self is remarkably flexible (e.g., Brugger and Lenggenhager, 2014; Clark, 2003, 2007; Martel et al., 2016; Vignemont, 2011). On a social and communicative level, humans express themselves through desired states of transformation (Sandberg, 2013). As technological capabilities grow, machines become more part of this self-transformation and how we experience (ourselves) (Ihde, 1979). Modern technology, in unprecedented ways, promises to fulfill the romantic fantasy of radical (cybernetic) self-transformation (Coeckelbergh, 2017). Human Enhancement is a powerful method to induce changes to the narrative identity of humans (DeGrazia, 2005). Furthermore, the transformation of our bodies can influence how we experience our individual life stories and selves. Perception of our autobiographical stories reflects the world we live in (McAdams, 2001), and this world is shaped by deliberate actions carried out by technology, influencing our “soft selves” and vice versa (Clark, 2003; Heersmink, 2018). Adopting the notion of a malleable self, especially in its narrative function and closely tied to our mind and the environment, as proposed by the seminal work of Clark (2003): How does this aspect relate to the various means of Human Enhancement and their function of adapting ourselves instead of the environment?

At its core, the self denotes the agent’s recognition of itself as a unity, essential for effectively orchestrating actions (Barandiaran et al., 2009). If self-referential to the user’s self-efficacy (Bandura, 1997) and self-concept, Human Enhancement can change the notion of our assumed capabilities in a given situation. In this case, when engaging in Human Enhancement, *someone* equipped with a particular set of skills deemed insufficient, adapts *to something* due to imposed demands *on someone*.

What determines the transformation of the narrative “soft self” (Clark, 2003)? Related alterations seem to depend at least partially on the enhancement’s transformational effect. Gilbert et al. (2017) interviewed Parkinson’s patients with a deep brain stimulation implant. Patients reported a brain stimulation-induced feeling of “self-estrangement” in dependency to experienced alienation by their disease. This estrangement was expressed in a feeling of loss of control, respectively distorted perception of capacities. In another study, Kögel et al. (2020) conclude that restorative brain-computer interfaces are important in recovering or maintaining the sense of agency and social participation and can positively impact users’ self-image. The contingency on the enhancements effect is further shown by Ram et al. (2019), who reported that the self-efficacy for learning and performance did not differ between students who used cognitive enhancement drugs and those who have not. This finding is understandable if we consider the limited effects of these substances (Repantis et al., 2010; Roberts et al., 2020), which may have failed to induce a sustainable change of self-concept and a failed test-in-the-world in the long run.

Hence, the extent of self-transformation seems to be linked to the perception of enhancement effects, especially in contrast to the pre-enhancement self. We are re-encountering a continuum here. Genetic engineering may have a more significant effect on self-conception than using cutlery. But at the same time, the ability to craft a meaningful personal narrative around the use of the technology may be more important for mitigating potential self-alienation than its objective disruptive potential (Iftode et al., 2022). Through Human Enhancement and the subsequent self-evaluation, I can experience myself and the world around me differently, even if only for a short period of time. The transformation of experience through the enhancement may occur not by direct sensorial augmentation but by changing the experience of being me. It does not change how we experience something; rather, it changes how we experience ourselves while we are experiencing something (Gilbert et al., 2017).

When used to adapt ourselves, means of Human Enhancement certainly extend aspects of our selves. Objects previously located *outside* now become *us*. This stresses the dynamic of constantly negotiating the boundaries between the agent and the environment (Clark, 2007). Although historically practiced, Human Enhancement may have been able to transform but not eradicate the self. The undeniable sense of self depends on the reflective understanding of physical capabilities and is likewise impacted by technological interventions (Clark, 2003). We are experiencing an extension and no dissolution of the socially operative self precisely because humans can maintain this essential psychological structure while undergoing repeated transformations on the narrative level (Clark, 2003; DeGrazia, 2005). The only thing that dissolves is the notion of a perceivable essential self that persists independently of the environment (Clark, 2003). The dynamic self may be constructed around a somewhat hard and stabilizing kernel. Yet, self-perception and social engagement tend to ignore this a priori condition and only acknowledge the surface phenomena.

One possible rationale for the preservation of the hard kernel is to uphold the living system’s integrity while also actively engaging in participatory social interactions (Maturana & Varela, 1987). The dynamic and “soft” self, assembled and transformed by various sources, operates within this social sphere (Clark, 2003). In addition, the environment encompasses other agents whose mental states remain beyond our reach. This transcendental aspect of the Other creates a fundamental separation between ourselves and the environment, laying the groundwork for relationships that may call for transformation and adaptation (Coeckelbergh, 2013).

Human enhancement reinforces the view that subjectivity and objectivity, in terms of what we experience as ourselves and what we experience as belonging to the world, are shaped by technology (Verbeek, 2005, 2014). Thus, the effects of respective means may be used to determine the borderline cases of self-environment distinction and inform us about this flexible boundary. Nonetheless, we must acknowledge the practical significance of the illusion of an encapsulated self on the level of effective social behavior. This encompasses seeing the enhancements someone uses as belonging to this person (Grewal et al., 2020) as playing an important part in their narrative self (Clark, 2003). As long as we maintain the belief that either *ourselves* or *someone* else must adapt to the environment and act accordingly, the self-environment distinction persists. This also holds also as long we put ethical meaning into the self-other distinction; as long as we affirm or reject something, including Human Enhancement, because it may affect ourselves in desired or feared ways, we bring the operational distinction into being.

Following Heidegger, the experience of reality is only possible through relation to it (Verbeek, 2005). The human-world relationship is mediated by different technologies (Ihde, 1990; Verbeek, 2008). Subsequently, different perceptions of and relations with the world will lead to a different reality and expectations of it (Carbon, 2015; Ramstead et al., 2016; Verbeek, 2005). Relation yields valuation, and to value means to relate (Coeckelbergh, 2013). Different aspects of our self represent different relationships and valued entities (Neisser, 1988). Hence, the experience of a world molded by technology is intrinsically linked to our perception and appraisal of ourselves. Through Human Enhancement, we transform our bodies and experiences and – depending on the extent of transformation – the notion of what it means to be ourselves in a given situation (Agar, 2014).

Still, we must acknowledge how the individual self is embedded in and constituted by the larger socio-material sphere. Even though an enhancement is, at foremost, a transformation of the individual, it may, again in the spirit of a cumulative enhancement effect, slowly change the notion of being human or being itself. What it means to be human is informed by many sources, including our assumed capabilities (Clark, 2003). By utilizing self-transforming technologies and operating within an environment, the modifications in our abilities or social behavior can lead to indirect downstream effects that would not have been possible if we had not chosen to adapt ourselves. Given the ubiquity of Human Enhancement and their increase in transformational power, “broader narrative contexts” may be necessary to maintain the ability to craft a meaningful narrative identity (Iftode et al., 2022) and make sense of the microlevel perception. Likewise, we should ask ourselves: *How will the change in our experience and the altered relationship with the world affect our selves?*

### What can be an Enhancement?

Ihde (1990) introduced the notion of technological *multistability*. Put very simply, this means that there is no essence of a particular technology but that it “can be used for a variety of purposes (the “multi”), but not for infinitely many (the “stability”)” (Boer, 2021, p. 1)^13^. Multistability acknowledges how different technologies can be used differently than intended and become an enhancement. Even, an intervention strictly employed in a therapeutic setting can still yield unintended yet controversial outcomes (Greely, 2006)^14^. The dominant mode of using a technology emerges from practice and is related to which possibilities of action the environment and artifact provide to the user (Boer, 2021). Therefore, multistability is tied to determining a technology’s moral value through related affordances (Boer, 2021; Klenk, 2021; Tollon, 2021).

Take Modafinil, a drug often used for cognitive enhancement purposes. This substance can improve chess performance under certain circumstances (Franke et al., 2017). Moreover, Modafinil’s enhancement effect was found to be present solely in participants with average IQs (Randall et al., 2005). For cognitive enhancement, the enhancement effectiveness, expectations, and eventual use may be linked to individual characteristics, as the relationship between drug dose and the corresponding effect may follow an inverted U-shaped curve tailored to the individual’s baseline (Husain & Mehta, 2011). Napoletano et al. (2020) identified a total of 142 unique substances that can be classified as cognitive enhancement drugs. The broadness of Human Enhancement and the dynamic world demands attention beyond means explicitly labeled as such and toward unexpected effects. After all, Modafinil has also been shown to increase the time to ejaculation in men with ejaculatio praecox (Haghighi et al., 2021). We should not forget that the variability of tasks and situational demands in relation to the technologies’ effects and individual capabilities determines whether the technology will live up to the enhancement motivation and pass its test-in-the-world.

The hermeneutic classification and adoption of technology as Human Enhancement hinge not solely on its initial design but also on the intentions when used in practice. These intentions are shaped by evaluating the technology’s effects against the practical and intentional backdrop and the technology itself (Verbeek, 2014). The technology’s affordances and our engagement with it and the world will determine its moral standing and reciprocal influence its use.

Consider genetic engineering via the notorious CRISPR/CaS9 technology. Even when upholding the treatment vs. enhancement distinction and not classifying therapeutic interventions as Human Enhancement, the case gets more complex when considering gene editing in the spirit of prevention (Juengst et al., 2018). While CRISPR is already in use for therapeutic reasons (Gillmore et al., 2021; Khalaf et al., 2020), the application of this technology to edit the genome of two twins to prevent infection with HIV sparked worldwide outrage (Almeida & Diogo, 2019; Cyranoski & Ledford, 2018; Wang et al., 2018). We can see a similar pattern of ethical evaluation based on intention when discussing invasive deep brain stimulation for enhancement instead of therapeutic reasons (Kostick-Quenet et al., 2022; Sattler & Pietralla, 2022). However, in other cases, the means employed may carry more weight than an intervention’s intended or factual outcome (Caviola & Faber, 2015; Haslam et al., 2021; Scheske & Schnall, 2012).

An illustrative example may be how wearing socks over shoes can enhance the human capability of maintaining stability on icy roads. The randomized control trial of Parkin et al. (2009) is an excellent case study of our notion of Human Enhancement. It showed how participants in the intervention group reported decreased slipperiness and how this self-assessment was successfully tested in the world (*r*_*observer rated−self rated slipperiness*_ = 0.7, the only participant who fell, was in the control group). At the same time, participants reported adverse side effects like a feeling of indignity when putting socks over their shoes. We do not claim that human gene editing and wearing socks over shoes carry the same ethical gravity. Yet, we want to emphasize that our technological lifeworld is full of potentially embodiable technology that can be used for enhancement purposes. Therefore, we should maintain an ethically attentive perspective that goes beyond technologies explicitly designed as enhancements.

After all, the phenomenon of multistability displays the creative potential of human material engagement. Thus, we should keep an open mind to identify potential enhancement opportunities afforded by conventional technologies and ask: *Could this technology become a means for Human Enhancement, and if yes, for whom?*

### On the Interplay Between Individual and Society

Cumulative material culture and technological developments are a complex and entangled process of material, individual, and social factors (Dobres & Hoffman, 1994; Jeffares, 2010; Malafouris, 2013; Morgan et al., 2015; Osiurak et al., 2016; Tennie et al., 2009), and Human Enhancement is no exception. Once developed, the presence of technology shapes social interaction and perception (Jeffares, 2010). Due to their intimate nature, Human Enhancement technologies have the potential to be conceived as part of someone and can thus play a vital role in the social influence agents exercise (Grewal et al., 2020).

#### Pull and Push Effects

As small and individual effects have the potential to aggregate into population effects (Almeida & Diogo, 2019; Powell, 2015), we must recognize potential cumulative effects, spilling over to higher levels of systemic organization in unpredicted trajectories (Allenby & Sarewitz, 2011). Collective enhancement always starts with a bottom-up process. The enhancement of a species is the emergent result of individual enhancement. At the same time, top-down research, financing, or regulation processes influence any major species-related enhancement efforts. These processes include social coercion to get enhanced but also a task-specific obligation to use enhancements (e.g., Allhoff et al., 2010; Elliott, 2011; Greely, 2006; Heinz et al., 2012; Hotze et al., 2011; Menuz et al., 2013; Pustovrh et al., 2018; Santoni de Sio et al., 2014; Sattler et al., 2022; Szocik, 2020). Social coercion and moral obligation to enhance are already occurring and have manifested in social practices in ways that seem quite “natural.” Cultural norms in many societies dictate the use of clothing and beauty products to conform to beauty or social conventions. Furthermore, an obligation to enhance may be present when embodied technologies are necessary to reach a certain performance level. It is improbable today that a hospital would hire a surgeon who vehemently refuses to work with surgical inspection enhancements such as binoculars. Recognition and distribution of technologies can yield new descriptive social, procedural, and ethical norms (Coeckelbergh, 2018; Verbeek, 2011) and *co-produce* the social order in which they are realized (Harbers, 2005b). This directly touches upon the evaluative criteria that determine to which degree the test-in-the-world is passed. Therefore, Human Enhancement technologies and practices must continuously be assessed concerning the social and institutional network from which they emerge (Allenby & Sarewitz, 2011).

Pressure that turns into a *pull* effect is just one side of the medal. The other is a *push* effect, which leads people to reject widespread Human Enhancement passionately (see Greely, 2006). Depending on the enhancement, people might define themselves over non-engagement with a particular enhancement. Some are already coquetting with not having a smartphone or even achieving specific tasks without technological help. Besides the aforementioned ethical and side effects-related reasons, people may also reject Human Enhancement due to concerns about the human body’s and mind’s immaculateness (Döbler & Carbon, 2021; Haslam et al., 2021; Mihailov et al., 2021). Or, more broadly: To minimize the potential of technological mediation of the “pure” world experienced by a “pure” body and mind (see Kass, 2003).

Under the premise of ubiquitous Human Enhancement exercised to adapt ourselves to a dynamic environment further and the general technological mediation of our experiences, this pureness must remain an illusion (Ihde, 1990). Yet, this does not mean that people will abandon the desire for what they conceive as “pure” experience and resist certain transformations deemed unbearable. Even when rejection of specific means may appear irrational: The individual gut feeling can exert a compelling influence, either attracting or repelling individuals from embracing a particular enhancement. Such individual resistance should serve as a reminder that the right way of adaptation is not self-evident and that ethical considerations should always inform instrumental arguments.

#### Adapting to the Social-Economic Environment

Levy (2012), arguing for the selective adaptation of our environment, questions whether adapting ourselves is the best strategy for assisting individuals in achieving their objectives effectively. Indeed, we should approach the individual and social desire for enhancement from two sides. The reasons for the perceived inadequacy of an individual’s current capabilities relative to experienced demands should be examined, before solely focusing on the potential risks or benefits associated with pursuing enhancement.

To become a “better” human – against what motivational and task-specific backdrop whatsoever – requires committing to a notion of a relatively “bad” human. “Bad” can refer to a maladaptive condition in relation to imposed demands – someone who is not adequately adapted. Just as wearing shorts to a dinner party would be considered a “bad” choice. Thomas (2022) argues that the idea of a contemporary “bad” human is implicitly dehumanizing and may serve as a justification for advocating radical enhancement. As he links his arguments directly to capitalism, we acknowledge how the predominant socio-economic system has installed a normative primacy of productivity, which individuals must adapt to. Engagement within this system is influenced by the ideological idea of a neoliberal “Entrepreneurial self” under the “growth imperative” (G. Adams et al., 2019). Although neoliberalism often appeals to the individual’s responsibility, the world to which one adapts is enriched by alien and dynamic needs and demands. Once we are thrown into this world with all its social-material contingencies, valuation emerges from the relation that inevitably occurs (Coeckelbergh, 2013). It is precisely this valued relationship, maybe in the form of feeling overly responsible to an employer (Pustovrh et al., 2018), that can lead to morally and socially reprehensible reasons for enhancing oneself.

Note, for instance, how individual positive performance effects of enhancement at the workplace can negatively spill out into personal life (Pustovrh et al., 2018). Accordingly, cognitive enhancement can lead to adverse work expectations by the employer, and even the self-determined motivation to enhance can be quickly swallowed by capitalist market dynamics. Using enhancement due to a feeling of responsibility for their employers may clash with the employee’s responsibility for personal health (Pustovrh et al., 2018).

Technologies can confront us with a mediated image of ourselves, leading to a feeling of responsibility and subjectification (Bergen & Verbeek, 2021). This phenomenon may be further accelerated by the increasing availability of technologies that enable individuals to measure and display their bodily processes. The subsequent creation of a “laboratory of the self,“ which fosters self-discovery and reveals potential areas for improvement (Kristensen & Ruckenstein, 2018), may incentivize individuals to pursue enhancement efforts.This can be further advanced by a society that has embraced neoliberal virtues. Even if we do not use a particular enhancement, we are still socially exposed to the general possibility. In neoliberalism: “The worker provides a skill set that can be enhanced according to the employer’s requirements—part of what is being offered is the worker’s reflexive ability to be an improvable subject” (Gershon, 2011, p. 540). Note how the social world conveys the responsibility to get enhanced; to change your relationship with it, or to improve your grip. Within this enterprise, individual enhancement effects provide the prospect of a better and more efficient collective future. Regardless of whether this is objectively necessary, the mere possibility of improving can lead us to experience ourselves as unsuitable. If not enhanced, we may experience a misfit, a loosened grip on the situation due to an increased disequilibrium that must be reduced. This is comprehensibly shown by Franke et al. (2013). Their survey among surgeons reports that work-related and personal-life performance pressure increases the probability of engaging with illicit and prescription cognitive and mood enhancement drugs. Furthermore, individuals high in stress deemed certain types of cognitive enhancement as more morally acceptable and were more willing to use it (Sattler & Pietralla, 2022). Experienced demands can predict the use of illicit means to boost cognitive performance (Wolff & Brand, 2013).

The dynamic and ever-changing nature of the environment-self system makes the pursuit of an optimal grip a Sisyphean task (Rietveld et al., 2018). This is symptomatic of the constantly changing capitalist social-material environment. Capitalism fuels crises and demands constant adaptation and overcoming of self-constructed hurdles (Žižek, 1989/2008, p. 53). The neoliberal subject is confronted with allegedly free choices related to individual self-fulfillment and radical self-authorship, which, in truth, constitute implicit demands instead of authentic empowerment (Adams et al., 2019; Žižek, 2019)^15^. How does one meet the responsibility of caring for themselves in the face of constant socioeconomic circulation? By improving one’s capabilities to adapt to the social-economic environment! These adaptations comprise enhancement techniques such as a neoliberal version of mindfulness (Purser, 2015). However, as a last resort, the neoliberal subject’s body becomes a suitable object for technological enhancement as well.

Our arguments seek to underscore the centrality of the socio-material and economic landscape in shaping the utilization and interpretation of emerging technologies (Coeckelbergh, 2013, 2018; Harbers, 2005a; Ihde, 1990; Thomas, 2022), how such utilization can be symptomatic of deeper systemic issues, and consequently, the self-immunizing character of a system that champions individual adaptation over modifying the socio-material environment.

Technology answers the moral question “How to live?” (Verbeek, 2005, p. 236). Human Enhancement, predominantly in its most radical forms, confronts us with the additional questions of what humans we want to be (Clausen, 2016), how we want to shape our lives (Coeckelbergh, 2011), and how to make sense of our bodily experiences in a techno-scientific world, in which we are able to measure and interpret every aspect of our biological foundation (Schmidl, 2022). While heaving an undeniably individual component, Human Enhancement must also be seen in the light of the socio-economic context. Adapting ourselves instead of the environment is made possible by the flexibility of our bodies, capabilities, practices, customs, and moral values, which make survival in an ever-changing environment possible in the first place. We are not static but perforce dynamic and affective beings. Our social values change, and they are changed by technology and science (Carroll, 2017; Ihde, 1990; Verbeek, 2011). Thus, values may differ from generation to generation, dependent on the grade of technological development, social context (Bostrom & Roache, 2008; Coeckelbergh, 2013; Daniels, 2000), and environmental demands. The challenge is determining the values worth preserving (Davies, 2017).

With the rapid advancement of technological capabilities, two crucial questions call for answers more than ever: *How will an enhancement impact society now and in the future?* And: *How much am I told to get enhanced by whom?*

## Being Human Through Old and New Technologies

Swift progress in the NBIC may substantiate the impression that Human Enhancement is a futuristic issue whose development demand either additional acceleration or preventive caution. However, *Homo sapiens* and their ancestors have continuously enhanced their capabilities through technology (Allenby & Sarewitz, 2011; Buchanan, 2011; Greely, 2006) and incorporated externalities into their mental workings (Clark, 2003). Largely building on previous works by Clark (2003), Ihde (1990), and Verbeek (2005), we have extended the discussion on Human Enhancement and included everyday technologies such as cutlery and clothes. Our approach does not seek to equate the ethical significance of wearing jeans and genetically modifying humans. Rather, the aim is to delve into the core functional aspect of Human Enhancement that underpins the continuum from mundane enhancements to their most radical futuristic applications. By doing so, we acknowledge different enhancements in different contexts, leading to varying transformations of experience and different ethical evaluations. Instead of being subjugated by the general fear of a dystopian, body-less posthuman future, we should be more attentive to the precise transformative effects of emerging novel bio-technological compositions (Clark, 2003).

Some may accuse us of overextending the notion of Human Enhancement, as the ethical debate about this issue is not about forks but technology that: “involves improving significant human attributes and abilities to levels that greatly exceed what is currently possible for human beings.” (Agar, 2010, p. 1), rendering not enhancement per se the issue, but surpassing of biological limits is (Agar, 2014). However, when assigning moral suspicion to Human Enhancement based on whether it can significantly improve biological boundaries, identifying the maximum for specific traits is difficult, if not impossible (Gyngell & Selgelid, 2016). Moreover, due to the cumulative and dynamic nature of human culture and technology, significant improvement of attributes is not novel. Human inheritance goes beyond genes, as each new individual also inherits the cognitive-technological niche and way of life established by previous generations (Coeckelbergh, 2018; Wheeler & Clark, 2008).

Is Human Enhancement not simply human technology, named differently? Some scholars resolved this issue by constraining Human Enhancement to “internal” technologies (e.g., Allhoff et al., 2010). However, “[i]f we insist on an internality constraint, as we must if the concept of enhancement is not to collapse into the concept of technology generally, then we face the problem of how to define such a constraint” (Bostrom & Roache, 2008, p. 121). As we do not wish to equate it with technology per se, we advocate seeing Human Enhancement as either psychologically and/or physically embodied technologies. This way, we join previous lines of argumentation on embodiment (e.g., Clark, 2003) with the contemporary Human Enhancement debate, which encompasses biotechnological interventions like drugs and genetic engineering and their controversial ethical implications. Put very shortly: Human Enhancement is doing technology to us.

This embodiment of the technology, in conjunction with the related transformation of experience, albeit no new phenomenon, is one reason for ethical unease regarding Human Enhancement. Leon Kass on biomedical enhancements:

“All of our encounters with the world, both natural and interpersonal, would be mediated, filtered, and altered. Human experience under biological intervention becomes increasingly mediated by unintelligible forces and vehicles, separated from the human significance of the activities so altered (Kass, 2003, p. 22)^16^

Why and at which threshold technological biomedical mediation – compared to the pervasive and ubiquitous technological mediation that also shapes and constitutes subjectivity and objectivity (Verbeek, 2005) – becomes unbearable is unclear. Still, Kass’ argument acknowledges our central premise: Augmenting our bodies, minds, capabilities, and skills, Human Enhancement can transform our individual experience (Agar, 2014) by altering our embodied relationship with the world. Human Enhancement may cause unease due to the prospect of changing the constituting factors of our existence on Earth and how we experience them: an artificial technological transformation of being itself.

Before the advent of vaccination, safely immunizing humans from dangerous diseases was incredibly hard. Without writing tools, humans could not materially preserve knowledge; without early stone tools like the hand axe, cutting and smashing raw materials was impossible (Döbler & Carbon, 2021; Greely, 2006). If the enhancement of capabilities itself threatens human nature (President’s Council on Bioethics, 2003), then we stopped being human before we even had the ability to write about what it means to be human. This is the logical implication of the argument that the experience of being human within a given world is brought forward through the ability to embody external things (Clark, 2003) and the subsequent technological mediation of experiences (Verbeek, 2005). Hand axe, hammer, spear, clothes, and vaccinations. These “conventional” technologies are helping humans act more efficiently, overcoming environmental challenges, increasing their grip, and potentially their well-being due to goal achievement and self-efficacy (Buchanan, 2011).

Agar (2014) acknowledges the experience-transforming potential of Human Enhancement and prospectively warns against radical enhancement that may undermine human values. In line with Clark’s proposals (2003), we align ourselves and extend the arguments, asserting that technologically embedded science in practice (Ihde, 1990, 2011) and the profound historical impact of technology, including Human Enhancement, have already significantly transformed our culturally transmitted top-down (Macro) model and understanding of both the world and ourselves (Clark, 2003; Ihde, 1990; Verbeek, 2005). The notion of our (technological) human being (Coeckelbergh, 2013) and our reality would be fundamentally different without the technological mediation of experience (Ihde, 1990, 2011), so would be the contemporary position from which we define which values to preserve.

**Fig. 3:**
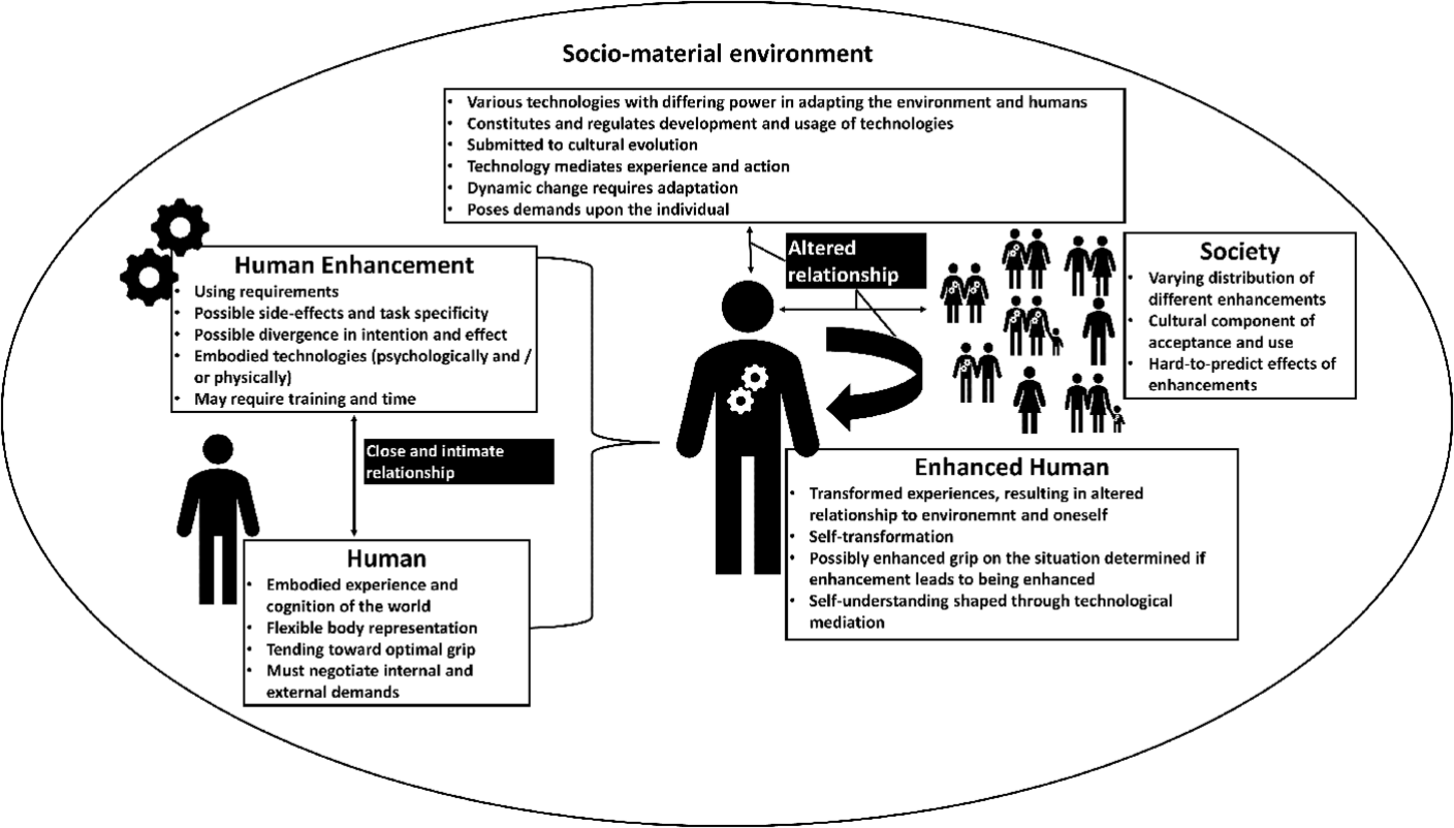
Overview of our approach

The influence of means for Human Enhancement reaches beyond the enumeration of now possible experiences but address and constitute the experiencing human subject. This is also true for external tools and their deep integration into our “mental profiles” that may ultimately “[…] impact who, what and where we are.” (Clark, 2003, p. 198). Being able to engage in various intimate relationships with technology that shape our practical engagement with the world, “Technologies […] are not opposed to human existence; they are its very medium.” (Rosenberger & Verbeek, 2015, p. 13). Situating the individual transformed experience within the cultural hermeneutic context (Ihde, 1990), *the experience-altering power of Human Enhancement does not threaten the image of human being per se but was and is part of its constitution!* Adapting ourselves rather than the environment targets one constitutive element of our world in relation to the other one. As technology, including Human Enhancement, co-shapes subjectivity and objectivity (Verbeek, 2005), and we are predestined to extend our soft selves through external means (Clark, 2003), can we criticize Human Enhancement for future radical change when the valued status quo is a product of the same radical function? (Fig. 3).

Human Enhancement is not a mere extension of human capabilities but rather a very influential and significant phenomenon in consciously changing one’s experience *of* and relationship *with* the world (Verbeek, 2005, 2008). Given the typical dynamic environmental demands, human-environment relations are in steady flux, the extent of which is also determined by the growing potential and pervasive presence of technology in general and Human Enhancement in particular. The relative degree of individual enhancement is influenced by humanity’s historical capacity to successfully cope with imposed demands (Cassioli & Balconi, 2022). The transformative potential of Human Enhancement technologies arises from their capabilities, our embodied relationship with the world, and the fact that Human Enhancement works close to, if not within, our bodies, blending these aspects at a hitherto unprecedented level. Mind, things, and bodies become even more “’*synechēs’* (continuous)*”* (Malafouris, 2013, p. 244). Due to its neuronal plasticity, the human brain adapts to the use of tools, altered social circumstances, and the new environments created with the help of technologies (Clark, 2003).

How we experience the world in and through our bodies influences our functional models of its workings, of what we can do, what we are, and what we will be able and will be (Clark, 2003; Döbler & Bartnik, 2022; Ihde, 1990; Rietveld et al., 2018). In the face of feared transformations, juxtaposed with the pervasive historical prevalence of Human Enhancement, we find ourselves questioning: *Is being human without Human Enhancement even possible?*

## Implications and Directions for Research and Development

The main objective of this article is to underscore critical elements in the Human Enhancement discourse while establishing a solid theoretical foundation that connects empirical observations with philosophical principles. By doing so, it seeks to enable well-informed decision-making concerning the implementation or regulation of these technologies. Therefore, we identified relevant questions as cornerstones for an evaluative case-by-case framework (Table 1). Although they may not be plenary, and their answers require extensive empirical testing and theoretical discussions, stakeholders can utilize them to start systematically evaluating Human Enhancement, its related phenomena, and the potential divergent effects.

**Table 1:** Relevant questions for a case-by-case evaluation

	Section	Questions
1	Is it always an improvement?	A) In which context does the enhancement benefit its users?
		B) What are possible individual (phenomenological) side-effects of the enhancement?
2	Testing Intention vs. Effect	C) How do intention and effect align?
		D) Will the enhancement pass the test-in-the-world and enhance its user?
		E) What are the relevant dimensions of evaluation?
3	What about the self?	F) How will the change in our experience and the altered relationship with the world affect our selves?
4	What can be an Enhancement?	G) Could this technology become a means for Human Enhancement?
		H) And if yes, for whom?
5	On the interplay between individual and society	I) How will an enhancement impact society now and in the future?
		J) How much am I told to get enhanced by whom?

Answering these questions will elucidate the complex relationship between the enhancement’s transformative capabilities at various levels and the possible ethical significance of it.

Our approach can inform concrete research endeavors that delve into the intricacies of human-technology interaction. Starting from the identified need to adapt to a certain demands, a significant emphasis should be placed on investigating the experience transformative capabilities of specific enhancements. This may enable a deeper comprehension of a technologies’ impact on human practices and its socio-material environment that are not captured by the designer’s intention.

Other researchers may also be inspired to investigate the tangible effects of contemporary and historical technologies through the lens of Human Enhancement. Such a comprehensive perspective may foster an understanding of strong attitudes toward specific technologies (Döbler & Carbon, 2021) while linking them to a general behavior that cuts deep into the history of our species. Besides the alignment with the bigger picture, a fruitful starting point for further psychological and social research investigating the different types of enhancement may adopt the questions concerning cognitive enhancement posed by Racine et al. (2021): Significance of outcomes, equivalence of methods, and concrete users motivation.

We are aware that theoretical considerations may have a difficult stand in the practical development of emerging technologies. However, specific technologies are far from being purely functional or even neutral. Instead, how they transform human experience and guide human behavior entails moral significance (Verbeek, 2011, 2014). Our framework may be suitable starting point for systematically analyzing the enhancement’s effect. Yet, it cannot answer whether the aspects transformed by the technology carry relevant value in a given context. Hence, research on the moral evaluation of Human Enhancement (e.g., Haslam et al., 2021; Maier et al., 2015; Scheske and Schnall, 2012) should be combined with approaches like ours to assess which moral values are important and how specific technologies endanger them. Studies should focus on encompassing cultural and individual diversity to assess possible axiological differences and different understandings of what constitutes a successful test-in-the-world. The significant economic interest behind the development of certain enhancements shall not serve as an excuse to accelerate the development, but as a reason for deep contemplation about the ethical meaning of transforming human capabilities in the intended way.

Hence, we recommend that research and development departments and executives of emerging technologies carefully contemplate how their technologies may impact the relationships they entail, including the one we have with ourselves: the very notion of being human.

## Enhancement Zero?

Stanley Kubrick’s “2001 – A Space Odyssey”, written together with Arthur C. Clarke (Kubrick, 1968), displays how early *Hominini*, influenced by a mysterious black monolith, discovered how a bone affords to be used as a weapon. The famous match cut links this *Tool Zero* directly to the human conquest of space.

Leaving the realm of science fiction, Human Enhancement, or more precisely, Hominin Enhancement, may have started once the respective species were able to use tools in a systematic, sustainable, and strategic way. The oldest stone tools date back ~ 3.3 Mya (Harmand et al., 2015), but the capacity to use tools may have also been present in earlier hominins (Lewis & Harmand, 2016; Panger et al., 2002). These early (stone) tools were the first enhancements that brought forward a new world. Operated directly by an agent: “Tool use offers new possibilities for cognitive extension. It affords new forms of embodied praxis, and thus opportunities for the development of the experience of agency and self-awareness” (Malafouris, 2013, p. 233).

The precise nature of our ancestors’ phenomenological embodied experience during tool-making and tool use, as well as the conscious intent behind the initial act of picking up the first tool, remain uncertain and speculative. Still, we argue that using these early tools, especially the first knapped hand axes – manufactured in embodied practice (Malafouris, 2013) – enhanced the capacities of body and mind and led to the transformation of experiences and an altered relationship with the world. These tools paved the way for humanity’s non-linear but cumulative cultural history that has found its tentative peak in the development of NBIC (Nanotechnology, Biotechnology, Information technology, and Cognitive science) means and widespread and devastating environmental manipulations. However, since these prehistoric tools were also the first hominin/human technology, adapting ourselves instead of the environment may be the hurdle we must overcome to alter the environment globally.

## Conclusion

There is no reason to fear technology or Human Enhancement per se. Due to their everlasting and constituting presence and evolutionary role in our species’ survival, neither one nor the other can threaten an alleged human essence (Ihde & Malafouris, 2019). Rather, the notion of being human is not pre-given but builds on the ability, need, and outcomes of adapting to the environment *and* ourselves through technology. In fact, both strategies seem to be reciprocally linked: First, through the necessity to extract resources from the environment to manufacture enhancements. Additionally, successful adaptation may create new possibilities to adapt the environment more efficiently. Lastly, through active engagement with the environment and the utilization of our potentially enhanced skills, we initiate a dynamic process that necessitates further adaptations.

While the growing potential for environmental manipulation has led to disastrous outcomes, the potential of modern enhancements is undeniably rising. In preparation, we propose to broaden our perspective and examine the transformative potential of contemporary and historical enhancements and directly link them with ethical considerations. This way, we may learn important lessons about potential opportunities, pitfalls, false hopes, and irrational fears. Although adapting to our environment appears inevitable, we can discuss and determine the desired transformations in our relationship with the environment (Coeckelbergh, 2013) along the lines we have drawn in this article.

The fact that the target of the technological intervention is a human being usually comes with a firm default opinion on Human Enhancement. We think that these opinions seldom acknowledge the manifoldness of this phenomenon. Due to the strong contextuality and interindividual differences in their effects, an *a priori* attempt to define morally “good” or “bad” enhancements based on ambiguous terms or exclusive perspectives is disadvantageous.

The ability of an organism to adapt its environment to its demands is a powerful evolutionary strategy (Kirsh, 1996). Humans have exercised this strategy unprecedentedly, changing their cognitive niche and the evolutionary trajectory of themselves and their planet. But in the course of our cumulative culture, Human Enhancement has carved out a unique yet seemingly transparent role. These technologies enabled present and extinct members of the genus *Homo* and potentially its closest ancestors to change their environment and intervene with their “natural” abilities and capacities, enhancing them beyond “normal” parameters and even creating new ones. By doing so, humans are not adapting the environment but themselves to the environmental and other demands. The transformational effect of Human Enhancement has co-constituted our state of being a *human being*. Ihde and Malafouris (2019) have recently emphasized that engaging in creative material engagement sparks a dynamic self-reflective process where humans both shape and are shaped by the objects they interact with. Human Enhancement may be the best example of such *becoming-by-making-(us)*.

## Footnotes

1 Juengst (1998) discussed enhancement in a bioethical framework, suggesting that he may have had biotechnology in mind.

2 This statement’s applicability is limited when discussing embryonic genetic enhancement. Though our ideas could be extended to this scenario, it requires a more prospective perspective. A comprehensive examination of this topic and the ontology human embryos is exceedingly intricate but falls outside the scope of this paper.

3 Our notion of technology is not limited to material artifacts, otherwise software would not be qualified as such. Technology comprises products of knipping a stone as much as of the skillful manipulation of transistors on a CPU through syntactic commands. Normative application of an artifact, software, tool etc. and its fabrication belongs to the realm of technique, the end-product of material engagement, however, is technology.

4 See Clark (2003) for an extensive discussion on the neuroscientific evidence and implications of the embodiment of external tools. The work of Clark and his arguments were very influential for this paper, especially his comments on the “soft self” and the subsequent meaning for our understanding of what it means to be human. However, when discussion Human Enhancement we must account for more than external tools. Hence, we approach the different human-technology relations from the broader postphenomenological side and also try to incorporate research published past 2003.

5 This categorization may overlap with other human-technology relationships defined by Ihde (1990) and others.

6 The prime example would be cosmetic surgery.

7 Whether the enhancement is an improvement of an already existing capability or the creation of a new one is often unclear. Would the ability to communicate brain-to-brain by brain-computer interfaces be an improvement of communication abilities or is this the emergence of a new capability?

8 For a discussion of the CI example concerning Human Enhancement, see Lee (2016).

9 This particular definition also emphasizes the experience altering power of Human Enhancement.

10 The referring to “improvement” is also implied by the identified general purpose of using technology to adapt toward the environment. If both aspects would be stripped away, identification of technologies becomes arbitrary so that the only relevant aspect would be an somehow justified assessment of moral permissibility see Chadwick, 2009.

11 Although Menuz et al. (2013) call for an a-posteriori definition of Human Enhancement, their approach allows for a dynamic assessment. Yet, its main focus lies in the outcome of the intervention.

12 An exception to this is *enhancement by accident*. Employing a fictional example here, Peter Parker did obtained his super powers by conscious choice. Neither did the radioactive spider bite him on purpose. Still, Parker is enhanced. Discussion of these effects are important, yet outside of the scope of this paper. However, we believe that, besides the difference in intentionality, the majority of our thoughts can be applied to such examples as well.

13 Ihde (1990) also situates the microperceptual ambiguity into a range of compatible macroperceptual contexts.

14 It is because of these unpredicted effects, that we consider Human Enhancement as *semi*-targeted process.

15 Another feature of neoliberalism is a strong focus on affect management (G. Adams et al., 2019). Compare this to the prospect of experiencing enhanced emotions or control undesired ones through Human Enhancement (Bostrom, 2013; Persson & Savulescu, 2012).

16 ”This is quite a Heideggerian perspective, in which the possibility of reality (humans) as the object of technological exploitation is a catastrophe see Žižek (2021, p. 194).
